# Measuring implementation fidelity of school-based obesity prevention programmes: a systematic review

**DOI:** 10.1186/s12966-018-0709-x

**Published:** 2018-08-13

**Authors:** Rosanne Schaap, Kathelijne Bessems, René Otten, Stef Kremers, Femke van Nassau

**Affiliations:** 1 Department of Public and Occupational health, Amsterdam Public Health research institute, Amsterdam UMC, VU University Amsterdam, Van der Boechorststraat 7, 1081 BT Amsterdam, The Netherlands; 2 NUTRIM School of Nutrition and Translational Research in Metabolism, Department of Health Promotion, Maastricht University, PO Box 616, 6200 MD Maastricht, The Netherlands; 3 VU University Amsterdam, Medical Library, De Boelelaan 1117, 1081 HV Amsterdam, The Netherlands

**Keywords:** Implementation fidelity, School-based obesity prevention programmes, Adherence, Dose, Responsiveness, Quality of delivery, Differentiation, Adaptation

## Abstract

**Background:**

Until now, there is no clear overview of how fidelity is assessed in school-based obesity prevention programmes. In order to move the field of obesity prevention programmes forward, the current review aimed to 1) identify which fidelity components have been measured in school-based obesity prevention programmes; 2) identify how fidelity components have been measured; and 3) score the quality of these methods.

**Methods:**

Studies published between January 2001–October 2017 were selected from searches in PubMed, EMBASE, PsycINFO, CINAHL, Cochrane Library and ERIC. We included studies examining the fidelity of obesity prevention programmes (nutrition and/or physical activity and/or sitting) at school (children aged 4–18 year) measuring at least one component of implementation fidelity. A data extraction was performed to identify which and how fidelity components were measured. Thereafter, a quality assessment was performed to score the quality of these methods. We scored each fidelity component on 7 quality criteria. Each fidelity component was rated high (> 75% positive), moderate (50–75%) or low (< 50%).

**Results:**

Of the 26,294 retrieved articles, 73 articles reporting on 63 different studies were included in this review. In 17 studies a process evaluation was based on a theoretical framework. In total, 120 fidelity components were measured across studies: dose was measured most often (*N* = 50), followed by responsiveness (*N* = 36), adherence (*N* = 26) and quality of delivery (*N* = 8). There was substantial variability in how fidelity components were defined as well as how they were measured. Most common methods were observations, logbooks and questionnaires targeting teachers. The quality assessment scores ranged from 0 to 86%; most fidelity components scored low quality (*n* = 77).

**Conclusions:**

There is no consensus on the operationalisation of concepts and methods used for assessing fidelity in school-based obesity prevention programmes and the quality of methods used is weak. As a result, we call for more consensus on the concepts and clear reporting on the methods employed for measurements of fidelity to increase the quality of fidelity measurements. Moreover, researchers should focus on the relation between fidelity and programme outcomes and determine to what extent adaptations to programmes have been made, whilst still being effective.

**Electronic supplementary material:**

The online version of this article (10.1186/s12966-018-0709-x) contains supplementary material, which is available to authorized users.

## Background

To combat the worldwide childhood overweight and obesity epidemic, a large variety of healthy eating and physical activity promotion programmes targeting youth have been developed [1]. Schools are regarded as a suitable setting for obesity prevention programmes, as they provide access to almost all children, regardless of their ethnicity or socio economic status [2]. School-based obesity prevention programmes that target healthy eating, physical activity and sedentary behaviour seem promising in reducing or preventing overweight and obesity among children [1, 3, 4]. However, when those evidence-based programmes are implemented in real world settings, their effectiveness is often disappointing [5]. One of the reasons is that programmes are not implemented in the same way as intended by programme developers, which could be labelled as a ‘lack of fidelity’ or ‘programme failure’ [5]. On the other hand, some degree of programme adaptation is inevitable and may actually have beneficial effects [6]. A better understanding of implementation processes is important to determine if and when disappointing effects can be ascribed to programme failure.

Although there is growing recognition for conducting comprehensive process evaluations, most studies are still focused on studying programme effectiveness and process evaluations are often an afterthought [7]. We believe that it is not only important to evaluate if a programme was effective, but also to understand how a programme was implemented and how this affected programme outcomes starting in the early stages of intervention development and evaluation [8]. The literature distinguishes the following four evaluation phases: 1) Formative evaluation: evaluate the feasibility of a health programme, 2) Efficacy evaluation: evaluate the effect of a health programme under controlled conditions (internal validity), 3) Effectiveness evaluation: evaluate the effect of a health programme under normal conditions (internal and external validity) and 4) Dissemination evaluation: evaluate the adoption of a large, ongoing health programme in the real world [9, 10]. Although there are differences in the scope of each evaluation phase, process evaluations can support studies in each phase by providing a detailed understanding about implementers’ needs, the implementation processes, specific programme mechanisms of impact and contextual factors promoting or inhibiting implementation [8, 10].

One of the aspects captured in process evaluations is fidelity [5, 11–13]. Fidelity is an umbrella term for the degree to which an intervention was implemented as intended by the programme developers [11]. Measurements of fidelity can inform us what was implemented and how it was done as well as what changes were made to the programme (i.e. what adaptations), and how these adaptations could have influenced effectiveness [11]. Until recently many claimed that all forms of adaptation indicate a lack of fidelity and, therefore, a threat to programme effectiveness [11, 12, 14, 15]. However, there is increasing recognition for the importance of mutual adaptation between the programme developers (e.g. researchers) and programme providers (e.g. student, teacher, school director) [11, 16]. Mutual adaptation indicates a bidirectional process in which a proposed change is modified to the needs, interests and opportunities of the setting in which the programme is implemented [11]. Wicked problems such as obesity require that programme developers are open to (major) bottom-up input and adjustments without controlling top-down influence [17]. Hence, this implies the need to accurately determine whether programmes were adapted and to measure fidelity and relate this to programme outcomes [10].

Several frameworks have been proposed and applied to measure fidelity in health-promotion programme evaluations [11, 18, 19]. For example, the Key process evaluation components defined by Linnan and Steckler [19] and the Conceptual framework for implementation fidelity by Carroll et al. [18]. Yet, there is much heterogeneity in the operationalisation and measurement of the concept of fidelity [20]. While some focus on quantitative aspects of fidelity, such as the dose delivered to the target group expressed in a percentage of use, others focus on if the programme was implemented as intended, often also operationalised as ‘adherence’ [11, 12, 18, 21]. Moreover, methods to measure fidelity may vary significantly in quality. While some focus on the use of teacher self-reports conducted at programme completion, others focus on the use of observer data during the implementation period which appears to be more valid [11]. No standardised operationalisation and methodology exists for measuring fidelity, partially due to the complexity and variety of school-based obesity prevention programmes.

In 2003, Dusenbury et al. reviewed the literature on implementation fidelity of school-based drug prevention programmes spanning a 25-year period [11]. According to their review, fidelity can be divided into five components: 1) adherence (i.e. the extent to which the programme components were conducted and delivered according the theoretical guidelines, plan or model); 2) dose (i.e. the amount of exposure to programme components received by participants, like the amount or the duration of the lessons that were delivered); 3) quality of programme delivery (i.e. how programme providers delivered the programme components, for example the teacher’s enthusiasm, confidence or way of telling); 4) participant responsiveness (i.e. the extent to which the participants are engaged with the programme, for example their enthusiasm, their interest in the programme or their willingness to participate); and 5) programme differentiation (i.e. the identification of essential programme components for effective outcomes) [11].

Until now, there is no clear overview of how fidelity is assessed in school-based obesity prevention programmes. In order to move the field of obesity prevention programmes forward, we reviewed the literature to identify the current methods used to operationalise, measure and report measures of fidelity. Building on the conceptualization of the elements of fidelity by Dusenbury, the aims of this review are to: 1) identify which fidelity components have been measured in school-based obesity prevention programmes; 2) identify how fidelity components have been measured; and 3) score the quality of these methods.

## Methods

### Literature search

A literature search was performed by RO, RS and FvN, based on the Preferred Reporting Items for Systematic Reviews and Meta-Analysis (PRISMA)-statement, see Additional file 1. To identify all relevant publications, we performed systematic searches in the bibliographic databases PubMed, EMBASE.com, Cinahl (via Ebsco), The Cochrane Library (via Wiley), PsycINFO (via EBSCO) and ERIC (via EBSCO) from January 2001 up to October 2017. Search terms included controlled terms (MeSH in PubMed and Emtree in Embase etc.) as well as free text terms. We used free text terms only in The Cochrane Library.

The search strategy focused on search terms standing for the target setting (e.g. ‘school’), in AND-combination with terms for measures of fidelity (e.g. ‘adherence’ OR ‘dose’), the target group (e.g. ‘child’), and on health promotion interventions (e.g. ‘health promotion’ OR ‘program’). This search was enriched with OR-combination with terms for at least one energy balance-related behaviour (e.g. ‘sitting’ OR ‘physical activity’ OR ‘eating’) or obesity (e.g. ‘obesity’ OR ‘overweight’). The full search strategy for all databases can be found in the appendix, see Additional file 2.

### Selection process

The following inclusion criteria were applied: 1) a population of school-aged children (4–18 years), 2) a school-based intervention that prevents obesity (sitting, nutrition and/or physical activity), 3) at least one fidelity component of Dusenbury et al. [11] as an outcome measure, 4) evaluating fidelity with quantitative methods – i.e. questionnaires, observations, structured interviews or logbooks. The following exclusion criteria were applied: 1) a population of younger (< 4 years) or older children (> 18 years); 2) evaluating programmes that were only implemented after school time; 3) evaluating fidelity with qualitative methods only. Only full text articles published in English and all studies describing elements of fidelity were included, irrespective of the maturity of the study phase (i.e. Formative evaluation, Efficacy evaluation, Effectiveness evaluation and Dissemination evaluation) [9, 10]. Two reviewers (RS, FvN) independently checked all retrieved titles and abstracts and independently reviewed all selected full text articles. Disagreements were resolved until consensus was reached. Finally, the reference lists of the selected articles were checked for relevant studies.

### Data extraction

Data extraction was performed to identify which and how fidelity components were measured. In the data extraction information was collected on author, programme name, year of publication, country of delivery, programme characteristics, setting, target group, programme provider, theoretical framework and measures of implementation fidelity. We used the classification of Dusenbury et al. [11] to review measures of implementation fidelity in school-based obesity prevention programmes. For each measured fidelity component (i.e. adherence, dose, quality of delivery, responsiveness, and differentiation), we extracted the following data: definition of fidelity component, data collection methods and timing, subject of evaluation (i.e. student, teacher or school), a summary of the results (i.e. mean value or range) and the relation between a fidelity component and programme outcome. One reviewer (RS) performed the data extraction. Thereafter, the data extraction was checked by a second reviewer (FvN). Disagreements between the reviewers with regard to the extracted data were discussed until consensus was reached. Results are reported per study, this means that if two articles reported about the same study trial, we merged the results. Studies describing the same programme but evaluated in different study trials were separately reported in the data extraction tables. Moreover, if the study referred to another publication describing the design or other relevant information about the study in question, the additional publication was read to perform additional data extraction. Three different authors were contacted by email to ask for additional information on the data extraction.

### Quality assessment

The quality assessment was performed to score the quality of methods used to measure each specific fidelity component (i.e. adherence, dose, quality of delivery, responsiveness and differentation) for each study. Two reviewers (RS and FvN) independently performed the methodological quality assessment. Disagreements were discussed and resolved. Given the absence of a standardized quality assessment tool for measuring implementation fidelity, the quality assessment was based on a tool that was used in two reviews also looking at process evaluation data [7, 22]. The quality of methods was scored by the use of 7 different criteria (see Table 1). As a first step, we assessed if the 7 criteria had been applied to each of the measured fidelity components in each study. Per fidelity component, each criterion was scored either positive (+) or negative (−) (see Table 1). However, this was slightly different for the fidelity components adherence and quality of delivery. For those components, the score not applicable (NA) was applied on criterion number two (i.e. level of evaluation), as it is not possible to evaluate these fidelity components on two or more levels – i.e. only on teacher level.

**Table 1:** Criteria list for assessment of the methodological quality of fidelity components

Criterion	Fidelity component receives a positive score	Fidelity component receives a negative score
1. Model used for evaluation	If a theoretical framework or model for the evaluation was used and reported or referred to in the article.	If no theoretical framework or model was used for the evaluation.
2. Level of evaluation	If the fidelity component was evaluated on two or more levels (i.e. school director, teacher, student).^a^	If the fidelity component was evaluated on only one level (i.e. school director, teacher, student).^a^
3. Operationalisation of fidelity component	If the fidelity component was defined or operationalised.	If only the name of the fidelity component was provided and not further defined or operationalised.
4. Data collection methods	If two or more techniques for data collection were used (triangulation).	If only one technique for data collection was used.
5. Quantitative fidelity measures	If measurement of the fidelity component was performed with adequately described methods.^b^	If measurements of the fidelity component was not performed with adequately described methods.^b^
6. Frequency of data collection	If the fidelity component was measured on more than one occasion (e.g. pre, during after delivery).	If the fidelity component was measured on only one occasion.
7. Relation fidelity component and programme outcome assessed	If tested whether the fidelity component was related to programme outcomes.	If not tested whether the fidelity component was related to programme outcomes.

^a^only applied to dose, responsiveness and differentiation, as it is not possible to evaluate adherence and quality of delivery on two or more levels – i.e. only on teacher level

^b^adequate = sufficient information to be able to repeat the study

Secondly, based on the scores of the 7 criteria, a sum quality score (percentage of positive scores of the 7 criteria) was calculated for each fidelity component for each study, resulting in a possible score of 0% (7 criteria scored negative) to 100% (7 criteria scored positive). The scoring procedure was slightly different for the fidelity components adherence and quality of delivery. Adherence and quality of delivery scored not applicable on criterion number two. Consequently, for these two fidelity components a sum quality score was calculated on the basis of 6 criteria. Which means that these components received 0% if 6 criteria were scored negative and 100% if 6 criteria were scored positive. Finally, each fidelity component was rated as having a high (> 75% positive), moderate (50–75% positive) or low (< 50% positive) methodological quality.

## Results

**Fig. 1:**
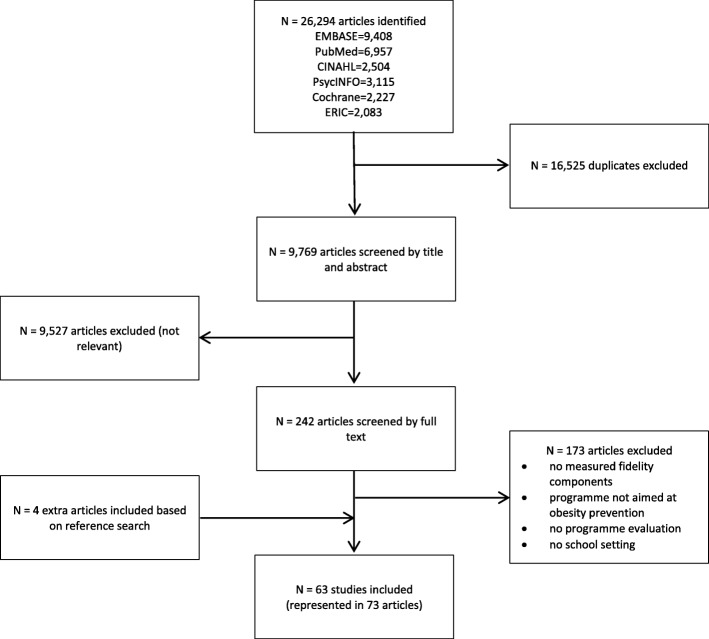
Flow diagram of study selection process

In total 26,294 articles of potential interest were retrieved: 9408 in EMBASE, 6957 in PubMed, 2504 in CINAHL, 3115 in PsycINFO, 2227 in Cochrane and 2083 in ERIC. After further selection based on first title and abstract and subsequently full text, 73 published articles [21, 23–94] reporting on 63 different studies were included. Reasons for excluding articles are reported in Fig. 1.

### Study characteristics

In the following section we will describe the study characteristics and describe which fidelity components have been measured in the included studies (Aim 1). Table 2 provides an overview of the study characteristics and measured fidelity components (i.e. adherence, dose, quality of delivery, responsiveness or differentiation) including references. Studies were conducted in 12 different countries, but were mostly conducted in the United States (*N* = 31) and in the Netherlands (*N* = 11). Two studies were conducted in a combination of multiple European countries. All studies were aimed to prevent obesity, wherein 16 studies targeted physical activity, 27 studies targeted healthy eating, 12 studies targeted both physical activity and healthy eating, three studies targeted both physical activity and sedentary behaviour and five studies targeted physical activity, healthy eating and sedentary behaviour. In total 45 studies were conducted in primary schools, 12 in secondary schools and six both in primary and secondary schools. The use of a theory, framework or model for designing process evaluations was applied in 17 studies. Nine different theories, framework or models were used; Key process evaluation components defined by Steckler and Linnan, Concepts in process evaluations by Baranowski and Stables, Conceptual framework of process evaluations by Mcgraw, How to guide for developing a process evaluation by Saunders, Logic model by Scheirer, RE-AIM framework by Glasgow, Probabilistic mechanistic model of programme delivery by Baranowski and Jago, Taxonomy of outcomes for implementation research by Proctor and Theory of diffusion of innovations by Rogers.

**Table 2:** Study characteristics

Study characteristics	Number of studies	References
Country		
• United States	*N* = 31	[21, 24, 26, 27, 29, 31–33, 36, 37, 42, 46–49, 53–57, 60, 61, 64–66, 69, 70, 75, 78, 80, 81, 85, 88–90, 93]
• Netherlands	*N* = 11	[28, 30, 38, 39, 44, 50, 52, 58, 62, 71, 72, 79]
• Australia	*N* = 4	[23, 34, 35, 83]
• Canada	*N* = 3	[40, 41, 63, 92, 94]
• United Kingdom	*N* = 3	[67, 68, 86]
• Belgium	*N* = 2	[82, 91]
• Norway	*N* = 2	[73, 74]
• Denmark	*N* = 1	[51, 77, 87]
• Finland	*N* = 1	[84]
• Germany	*N* = 1	[43]
• Pakistan	*N* = 1	[76]
• Sweden	*N* = 1	[59]
• Multiple European countries	*N* = 2	[25, 45]
Aim		
• Physical activity	*N* = 16	[26, 33, 36, 37, 39, 40, 50, 52, 57, 62, 73, 75, 76, 80, 83, 84, 91, 94]
• Healthy eating	*N* = 27	[24, 25, 27, 29, 31, 38, 42–45, 48, 51, 54, 55, 60, 63, 65, 67, 69–72, 74, 77, 79, 81, 85, 87, 88, 90, 92, 93]
• Physical activity and healthy eating	*N* = 12	[21, 23, 28, 30, 32, 40, 41, 46, 47, 49, 53, 55, 56, 59, 61, 64, 66, 76, 78, 82, 86, 93, 94]
• Physical activity and sedentary behaviour	*N* = 3	[34, 35, 73]
• Physical activity, healthy eating and sedentary behaviour	*N* = 5	[28, 30, 58, 68, 89]
Setting		
• Primary school	*N* = 45	[21, 24, 27, 29, 31, 34–36, 38, 40–43, 45, 48, 50, 52–55, 57, 60, 62–70, 73–76, 78–82, 85, 86, 88–93, 94]
• Secondary school	*N* = 12	[23, 26, 28, 30, 32, 33, 39, 44, 51, 58, 71, 72, 77, 83, 84, 87]
• Primary and secondary school	*N* = 6	[25, 37, 46, 47, 49, 56, 59, 61]
Theoretical framework underlying process evaluations	*N* = 17	[26, 28, 30, 33, 36, 46, 48, 51, 52, 56–58, 60–63, 77, 80, 86, 87, 91, 92]
Fidelity components	*N* = 120	
• Adherence	*N* = 26	[24, 25, 28–30, 32, 35–38, 44, 46–49, 51, 56, 59–61, 77, 81, 85–87, 90]
• Dose	*N* = 50	[21, 23–29, 31, 33–36, 38–41, 43–46, 48, 50–52, 54–58, 60–68, 70–80, 82, 83, 87, 89–93, 94]
• Quality of delivery	*N* = 8	[28, 32, 36, 57, 63, 75, 81, 89, 92]
• Responsiveness	*N* = 36	[23, 25, 28, 30, 32, 34, 35, 38–40, 42, 44, 46, 48, 51–53, 56–58, 63, 66, 67, 69–72, 74–77, 79–84, 87–89, 92, 94]
• Differentation	*N* = 0	
Number of fidelity components per study		
• One component	*N* = 24	[21, 26, 27, 31, 33, 37, 41–43, 45, 47, 49, 50, 53–55, 59, 62, 64, 65, 73, 78, 84–86, 88, 91, 93]
• Two components	*N* = 22	[23, 24, 29, 30, 34, 39, 40, 52, 58, 60, 61, 66–69, 74, 76, 79, 80, 82, 83, 90, 94]
• Three components	*N* = 16	[25, 32, 35, 36, 38, 44, 46, 48, 51, 56, 57, 63, 70–72, 75, 77, 81, 87, 89, 92]
• Four components	*N* = 1	[28]
• Five components	*N* = 0	

In total, 63 studies reported 120 fidelity components. Dose was measured most often (*N* = 50), followed by responsiveness (*N* = 36), adherence (*N* = 26), and quality of delivery (*N* = 8). The fidelity component differentiation was not measured in any of the studies. In 24 studies only one fidelity process component was measured. Two components were measured in 22 studies and three components were measured in 16 studies. Only one study measured four fidelity components, meaning that no study measured all five fidelity components.

In the following sections, each of the measured fidelity components are described in more detail. We describe how fidelity components were measured (Aim 2) and the quality scores for these methods (Aim 3). Tables 3 and 4 provide an overview on the measurements of the fidelity components including references. The full data extraction and quality assessment can be found in the appendix, see Additional files 3 and 4.

**Table 3:** Characteristics of methods used to measure fidelity component

Characteristics of methods	Number of studies	References
Adherence		
Data collection method		
• Observations	*N* = 15	[24, 29, 38, 44, 46–49, 56, 60, 61, 68–72, 86, 90]
• Logbooks	*N* = 10	[29, 35–37, 68, 70–72, 81, 85, 86, 90]
• Questionnaires	*N* = 7	[25, 28, 30, 32, 48, 51, 60, 77, 87]
• Structured interviews	*N* = 2	[47, 49, 59]
Subject of evaluation		
• Student	*N*/A	
• Teacher	*N* = 25	[24, 25, 28–30, 32, 35–38, 44, 46, 48, 51, 56, 59–61, 68–72, 77, 81, 83, 85–87, 90]
• School	*N* = 1	[47, 49]
Dose		
Data collection method		
• Observations	*N* = 7	[21, 26, 33, 38, 44, 48, 52, 64, 65, 75, 83, 89]
• Logbooks	*N* = 24	[21, 24, 26, 29, 33, 35, 36, 38–41, 44, 46, 50, 56–58, 62–64, 68, 70–72, 76, 78, 79, 89, 90, 92, 94]
• Questionnaires	*N* = 27	[21, 25, 27, 28, 31, 43, 45, 48, 50–52, 54, 55, 57, 58, 60–62, 64–67, 73, 74, 77, 80, 82, 87, 91, 93]
• Structured interviews	*N* = 6	[21, 34, 36, 43, 44, 50, 62, 64, 65]
Subject of evaluation		
• Student	*N* = 10	[21, 23, 27, 39, 51, 58, 64, 70, 73, 77, 82, 87, 90]
• Teacher	*N* = 40	[21, 24–26, 28, 29, 31, 33–36, 38, 40, 41, 43–46, 48, 50, 51, 54–57, 60–68, 71, 72, 74–80, 83, 89, 91–93, 94]
• School	*N* = 1	[52]
Quality of delivery		
Data collection method		
• Observations	*N* = 2	[32, 75]
• Logbooks	*N* = 3	[36, 81, 89]
• Questionnaires	*N* = 3	[28, 57, 63, 92]
• Structured interviews	*N* = 0	
Subject of evaluation		
• Student	*N*/A	
• Teacher	*N* = 8	[28, 32, 36, 57, 63, 75, 81, 89, 92]
• School	*N* = 0	
Responsiveness		
Data collection method		
• Observations	*N* = 5	[32, 46, 56, 57, 71, 72, 75]
• Logbooks	*N* = 3	[35, 71, 72, 89]
• Questionnaires	*N* = 34	[23, 25, 28, 30, 32, 34, 35, 38–40, 42, 44, 46, 48, 51–53, 56, 58, 63, 66, 67, 69–72, 74–77, 79–84, 87, 88, 92, 94]
• Structured interviews	*N* = 1	[44]
Subject of evaluation		
• Student	*N* = 31	[23, 25, 28, 30, 32, 34, 35, 39, 42, 44, 46, 48, 51–53, 56–58, 67, 69–72, 74–77, 79, 81–84, 87–89]
• Teacher	*N* = 17	[28, 30, 38, 40, 44, 51, 63, 66, 67, 69–72, 75, 77, 79, 80, 84, 87–89, 92, 94]
• School	*N* = 0

**Table 4:** Quality of methods used to measure fidelity components

Criterion	Number of studies	References
Adherence		
1. Model used for evaluation	*N* = 9	[28, 30, 36, 46, 48, 51, 56, 60, 61, 77, 86, 87]
2. Level of evaluation	NA	
3. Operationalisation fidelity component	*N* = 15	[28, 29, 35–38, 46–49, 51, 56, 59–61, 69, 71, 72, 77, 87]
4. Data collection methods	*N* = 8	[29, 47–49, 60, 68, 70, 86, 90]
5. Quantitative fidelity measures	*N* = 13	[25, 28, 36–38, 46–49, 51, 56, 60, 68, 71, 72, 77, 81, 87]
6. Frequency of data collection	*N* = 22	[24, 28, 29, 32, 35–38, 44, 46, 48, 51, 56, 60, 61, 68–72, 77, 81, 85–87, 90]
7. Relation fidelity component and programme outcome assessed	*N* = 5	[25, 28, 29, 46, 56, 81]
Methodological qualitsy per fidelity component		
• Low (< 50%)	*N* = 11	[24, 25, 30, 32, 35, 44, 59, 69, 70, 85, 90]
• Moderate (50–75%)	*N* = 11	[29, 36–38, 47, 49, 51, 61, 68, 71, 72, 77, 81, 86, 87]
• High (> 75%)	*N* = 4	[28, 46, 48, 56, 60]
Dose		
1. Model used for evaluation	*N* = 14	[26, 28, 33, 36, 46, 48, 51, 52, 56–58, 60–62, 77, 80, 87, 91]
2. Level of evaluation	*N* = 2	[21, 51, 64, 77, 87]
3. Operationalisation fidelity component	*N* = 28	[21, 23, 27–29, 34–36, 38, 46, 48, 50–52, 56–58, 61, 62, 64, 66, 71–73, 75–80, 87, 89]
4. Data collection methods	*N* = 13	[21, 26, 33, 39, 43, 44, 48, 50, 52, 57, 58, 62, 64, 65, 89]
5. Quantitative fidelity measures	*N* = 23	[25, 26, 28, 33, 36, 38, 39, 45, 46, 48, 51, 52, 56, 58, 60, 63, 65, 68, 71–75, 77, 79, 80, 87, 91, 92]
6. Frequency of data collection	*N* = 34	[21, 23, 24, 26, 28, 29, 33, 35, 36, 38, 40, 41, 44, 46, 48, 51, 52, 56–58, 62–68, 71–75, 77, 78, 80, 83, 87, 89–92, 94]
7. Relation fidelity component and programme outcome assessed	*N* = 17	[25–29, 33, 40, 45, 46, 51, 56, 58, 67, 71–74, 77, 79, 87, 89, 91, 94]
Methodological quality per fidelity component		
• Low (< 50%)	*N* = 34	[23–25, 27, 29, 31, 34, 35, 38–41, 43–45, 50, 54, 55, 60, 61, 63, 65–68, 70, 74–76, 78, 79, 82, 83, 90, 92, 93, 94]
• Moderate (50–75%)	*N* = 14	[21, 26, 28, 33, 36, 46, 48, 52, 56, 57, 62, 64, 71–73, 80, 89, 91]
• High (> 75%)	*N* = 2	[51, 58, 77, 87]
Quality of delivery		
1. Model used for evaluation	*N* = 3	[28, 36, 57]
2. Level of evaluation	NA	
3. Operationalisation fidelity component	*N* = 5	[28, 36, 75, 81, 89]
4. Data collection methods	*N* = 1	[89]
5. Quantitative fidelity measures	*N* = 5	[36, 57, 63, 75, 81, 92]
6. Frequency of data collection	*N* = 6	[28, 32, 36, 75, 81, 89]
7. Relation fidelity component and programme outcome assessed	*N* = 2	[28, 81]
Methodological quality per fidelity component		
• Low (< 50%)	*N* = 3	[32, 57, 63, 92]
• Moderate (50–75%)	*N* = 5	[28, 36, 75, 81, 89]
• High (> 75%)	*N* = 0	
Responsiveness		
1. Model used for evaluation	*N* = 10	[28, 30, 46, 48, 51, 52, 56–58, 63, 77, 80, 87, 92]
2. Level of evaluation	*N* = 14	[28, 30, 32, 44, 51, 67, 69–72, 75, 77, 79, 84, 87–89]
3. Operationalisation fidelity component	*N* = 13	[28, 34, 35, 44, 46, 48, 51, 52, 56, 66, 75, 77, 79, 80, 87, 89]
4. Data collection methods	*N* = 5	[32, 35, 46, 56, 75, 89]
5. Quantitative fidelity measures	*N* = 23	[23, 25, 28, 38–40, 42, 46, 48, 52, 53, 56, 58, 63, 69, 71, 72, 74–76, 79–81, 83, 84, 92, 94]
6. Frequency of data collection	*N* = 14	[28, 32, 35, 46, 52, 56, 57, 66, 67, 74, 75, 80, 84, 88, 89]
7. Relation fidelity component and programme outcome assessed	*N* = 7	[25, 46, 56, 58, 67, 74, 79, 81]
Methodological quality per fidelity component		
• Low (< 50%)	*N* = 29	[23, 25, 30, 32, 34, 35, 38–40, 42, 44, 48, 51, 53, 57, 58, 63, 66, 67, 69–72, 74, 76, 77, 81–84, 87, 88, 92, 94]
• Moderate (50–75%)	*N* = 6	[28, 52, 75, 79, 80, 89]
• High (> 75%)	*N* = 1	[46, 56]

### Adherence

The component adherence was reported in 26 studies (see Table 2). A definition of adherence was provided and defined or operationalised in 15 studies. Adherence was mostly defined as fidelity, programme carried out, delivered or intended as planned. Measurements of adherence were mostly conducted with observations (*N* = 15), followed by logbooks (*N* = 10), questionnaires (*N* = 7) and structured interviews (*N* = 2) (see Table 3). Teachers were in almost all cases the subject of evaluation, while schools (i.e. school leaders) were reported in one study. Regarding frequency of data collection, 22 studies measured adherence on more than one occasion (see Table 4). Adherence was related five times to programme outcomes.

Quality scores of adherence ranged from 17 to 83% and the mean score was 46%. Adherences scored 11 times low, 11 times moderate and four times high methodological quality (see Table 4). Adherence scored the highest on criterion six (i.e. frequency of data collection) and the lowest on criterion seven (i.e. fidelity component related to programme outcome), as in only five studies the relation between a fidelity component and programme outcome was assessed.

### Dose

The component dose was reported in 50 studies (see Table 2). A definition of dose was defined or operationalised in 28 studies. Dose was mostly defined as the proportion, amount, percentage or number of activities or components that were delivered, used or implemented. Measurements of dose were conducted by means of questionnaires (*N* = 27), logbooks (*N* = 24), observations (*N* = 7) and structured interviews (*N* = 6) (see Table 3). In one study the method was not reported [23]. In 40 studies, the subject of evaluation was a teacher, in ten studies a student and only in one study a school. Regarding frequency, 34 studies measured dose on more than one occasion (see Table 4). Dose was related 17 times to programme outcomes.

Quality scores of dose ranged from 0 to 86% and the mean score was 37%. Dose scored 34 times low, 14 times moderate and two times high methodological quality (see Table 4). Dose obtained the highest scores on criterion number six (i.e. frequency of data collection) and the lowest on criterion number two (i.e. level of evaluation), as in only two studies the level of evaluation was on two or more levels.

### Quality of delivery

Quality of delivery was measured in eight studies (see Table 2). Five of those studies that defined or operationalised quality of delivery, referred to it as the quality of the programme. For example, studies measured if teachers were prepared for their lessons, their way of communicating to children or their level of confidence to demonstrate lessons. Conducted measurements methods were questionnaires (*N* = 3), logbooks (*N* = 3) and observations (*N* = 2) (see Table 3). All studies were performed on teacher level and almost all studies, except for two, performed measurements on multiple occasions (see Table 4). Quality of delivery was related twice to programme outcomes.

Quality scores of quality of delivery ranged from 17 to 67% and the mean score was 46%. Quality of delivery scored three times low and five times moderate methodological quality (see Table 4). Quality of delivery obtained the highest score on criterion number six (i.e. frequency of data collection) and obtained the lowest score on criterion number four (i.e. data collection methods), because only one study reported the use of more than one technique for data collection.

### Responsiveness

Responsiveness was measured in 36 studies (see Table 2). The definition of responsiveness was defined or operationalised in 13 studies. Responsiveness was generally defined as satisfaction, appreciation, acceptability, or enjoyment of the programme. Measurements of responsiveness were mostly conducted with questionnaires (*N* = 34), followed by observations (*N* = 5), logbooks (*N* = 3) and structured interviews (*N* = 1) (see Table 3). The subject of evaluation was 31 times on student level and 17 times on teacher level. Hereby, measurements were in 14 studies on multiple occasions (see Table 4). Responsiveness was related seven times to programme outcomes.

Quality scores of responsiveness ranged from 0 to 86% and the mean score was 34%. Responsiveness scored 29 times weak, 6 times moderate and 1 time high methodological quality (see Table 4). Responsiveness scored the highest on criterion number five (i.e. quantitative fidelity measures) and scored the lowest on criterion number four (i.e. data collection methods), because only five studies reported the use of multiple data collection methods.

## Discussion

This review aimed to gain insight in the concepts and methods employed to measure fidelity and to gain insight into the quality of measuring fidelity in school-based obesity prevention programmes. The results of this review indicate that measurements of fidelity are multifaceted, encompassing different concepts and varying operationalisation of fidelity components. Moreover, methods were conducted in a range of different ways and mostly conducted with a low methodological quality.

The studies included in our review used different ways to define fidelity and its components in school-based obesity prevention programmes. One of the main concerns is that definitions of fidelity components were used interchangeably and were inconsistent. The same definition was used for different fidelity components. For example, adherence and dose were both defined as ‘implementation fidelity’. Definitions, if provided, were rather short and 59 of the 120 fidelity components were not accurately defined, meaning that only the ‘name’ of the component was mentioned and no further explanation was provided on how authors defined the measured component. This lack of consistency in fidelity components definitions is in line with other reviews in implementation science [11, 95] and may be due to the small amount of studies that base their process evaluation on a theory, framework or model [96].

Related to that issue, no standardised theory, framework or model exists for the guidance of measuring fidelity in school-based obesity prevention programmes [96]. As a result, process evaluation theories, frameworks or models from different research areas were implemented, which may also contribute to inconsistent fidelity component definitions. For example, Concepts in process evaluations by Baranowski and Stables originated from health promotion research [65], while the Taxonomy of outcomes for implementation research by Proctor originated from quality of care research [13]. Thus, researchers in implementation science need to agree upon the definitions employed for fidelity components, base their design for fidelity measurements on a theory, framework or model and describe this design and included fidelity components thoroughly.

According to Dusenbury et al. [11], fidelity has generally been operationalized in five components; 1) adherence, 2) dose, 3) quality of delivery, 4) responsiveness and 5) differentiation. In line with their findings, the current review showed that the amount and type of fidelity components measured in the included studies varied a lot, with most studies only assessing one to three components. Moreover, the majority of the studies only investigated dose, which reflects often primary interest of researchers in actual programme delivery and participation levels, rather than an interest in how a programme was delivered (i.e. quality of delivery). None of the studies measured the unique programme components, operationalized as differentiation by Dusenbury et al. [11]. One explanation could be that it is very complex to measure differentiation in school-based obesity prevention programmes, as they usually include many different interacting programme components which as a whole contribute to programme outcomes.

The finding that no study assessed every component of fidelity as defined by Dusenbury et al. [11] confirms previous findings [20]. Although, it can be argued that measurements of all fidelity components can provide a full overview of the degree to which programmes are implemented as planned [12]. This may often be unfeasible as the choice for which fidelity components to include in process evaluations is partially dependent on the context, resource constraints and measurement burden [10]. As such, measurements for fidelity are determined through both a top-down and bottom-up approach, which include programme developers, and programme providers. Additionally, the claim that measurements of all fidelity components is of importance, could also be debated by the fact that it is still unknown which of the fidelity components contributes most to positive programme outcomes. Subsequently, often too much focus is given on measuring as much fidelity components as possible, which could decrease the quality of fidelity measurements. Therefore, we may learn most from carefully selecting and measuring most relevant components with high quality. This may bring us one step further in learning more about the relation between fidelity components and positive programme outcomes.

Most components scored low methodological quality (i.e. 77 fidelity components). Especially methods employed to measure a fidelity component scored low. While measurements of fidelity components were performed with a wide variety of techniques (i.e. observations, questionnaires, structured interviews or logbooks), the majority of the studies lacked data triangulation and the employed methods were often not measured on two or more levels (i.e. teacher, student or school). A possible explanation could be that process evaluations of high quality may not always be practical to fulfil in real-world settings [97]. Herein, researchers need to balance between high quality methods and keeping the burden for participants and researchers low. Therefore, the complexity of school-based obesity prevention programmes may play a role in the quality of methods that are conducted in process evaluations [10]. Another explanation could be that many studies focus more on effect outcomes than on process measures. A multifaceted approach, encompassing both outcome and process measures, is needed as implementation of school-based obesity prevention programmes is a complex process [10]. Thus, we need to stimulate researchers to focus more on process measures encompassing more high-quality methods with a focus on data triangulation and measurements conducted on different levels.

Studies barely discussed the validity or reliability of measures used for measuring fidelity. This may be due to the fact that process evaluations are scarcely validated and are often adapted to the setting in which a programme is implemented. Identification and knowledge of strong validated instruments in the field of implementation science is limited. In a response to this lack of knowledge, the Society for Implementation Research Collaboration (SIRC) systematically reviewed quantitative instruments assessing fidelity [98]. Their review is a relevant and valuable resource for identifying more high-quality instruments. In addition, the SIRC also provides information on the use of these instrument in certain contexts, which is of importance for implementation research in real-world settings. Hence, to move the field of implementation science forward, future studies need to report in detail which methods were used for measuring fidelity and the inherent strengths and limitations of these methods and even more of importance is that journals support publication of these types of articles.

The importance for relating fidelity to programme outcomes in school-based obesity programmes is increasingly recognised [10, 20]. The majority of studies included in this review did not investigate the relation between components of fidelity and programme outcomes, which is in line with conclusions of other studies [20]. The investigation of this relation is of importance, as high fidelity simply does not exist in real practice; programmes that were adapted to the setting in which they were implemented (i.e. mutual adaptation) were more effective than programmes that were implemented as intended (i.e. high fidelity) [11]. More flexibility in programme delivery could address the needs of the target group or context in which the programme is implemented and may increase the likelihood that the programme will be adopted in real practice and, thereby, result in more positive outcomes [11, 99]. Though, process evaluations included in our review were often part of an RCT (i.e. implementation during controlled conditions) and were mainly focused on investigating the effectiveness of a programme as the main outcome. Randomisation is however often not desirable nor feasible in real world obesity prevention approaches [100]. Instead, more research is needed looking at the degree of implementation in real world settings and to what extent adaptations to the programme have been made, and how this impacted the implementation and sustainability of changes.

### Strengths and limitations

To our knowledge, this is the first study conducting a systematic review to provide an overview of methods used for measuring fidelity in school-based obesity prevention programmes. Other strengths are the use of the framework of Dusenbury et al. [11] to conceptualise fidelity, systematically select studies, data extraction and quality assessment performed with two reviewers independently. However, there are also some limitations of this review that should be acknowledged. First, formative evaluation or effectiveness evaluation studies may not have reported their process evaluations in the title or abstract, as implementation is rarely a key focus of school-based obesity prevention programmes. As a result, some relevant studies may have been missed. Nevertheless, we included a large number of studies, therefore, we assume that this review provides a good overview of fidelity in school-based obesity prevention programmes. Another limitation is the possibility that we have overlooked relevant data or misinterpreted the data, when conducting the data extraction and quality assessment. We tried to minimise this bias by having two researchers conducting the data extraction and quality assessment in order to obtain more accuracy and consistency, and authors were contacted for clarification, when needed.

## Conclusions

There is no consensus on the measurements of fidelity in school-based obesity prevention programmes and the quality of methods used is weak. Therefore, researchers need to agree upon the operationalisation of concepts and clear reporting on methods employed to measure fidelity and increase the quality of fidelity measurements. Moreover, it is of importance to determine the relation between fidelity and programme outcomes to understand what level of fidelity is needed to ensure that programmes are effective. At last, more research is needed looking at the degree of implementation in real world settings. As such, researchers should not only focus on top-down measurements. In line with mutual adaptation approaches in intervention development and implementation, a bidirectional process should be part of process evaluations, wherein researchers examine whether and under which conditions adaptations to the programme have been made, whilst still being effective and sustainable in real world settings.

## Additional files

Additional file 1: PRISMA checklist. PRISMA checklist wherein we indicated in which part of the manuscript each item of the checklist was reported. (DOC 64 kb)

Additional file 2: Search terms. Search strategy for various databases. (DOCX 29 kb)

Additional file 3: Data extraction. Full overview of the data extraction of the included studies. (DOCX 84 kb)

Additional file 4: Quality assessment. Full overview of the quality assessment of the fidelity components. (DOCX 48 kb)

## Abbreviations

NA: Not applicable
SIRC: Society for Implementation Research Collaboration
