# Supplementary material for: Measuring implementation fidelity of school-based obesity prevention programmes: a systematic review
Source: Int J Behav Nutr Phys Act. 2018 Aug 13;15:75. doi: 10.1186/s12966-018-0709-x (PMC6088402; doi:10.1186/s12966-018-0709-x)
Supplement: Supplementary file 2 — Search terms. Search strategy for various databases. (DOCX 29 kb) [file 12966_2018_709_MOESM2_ESM.docx]

**Additional file 2 - Search strategy**

**PubMed search strategy 2017 October 3^rd^**

| **#** | **Query** | **Results** |
| --- | --- | --- |
| **#11** | #10 AND "2001"[Date - Publication] : "2018"[Date - Publication] | **3924** |
| **#10** | #5 AND #6 AND #7 AND #8 AND #9 | **4697** |
| **#9** | "Schools"[Mesh:NoExp] OR school*[tiab] OR teacher*[tiab] | **266318** |
| **#8** | "Adolescent"[Mesh] OR "Child"[Mesh:NoExp] OR adolescen*[tiab] OR child*[tiab] OR teen[tiab] OR teens[tiab] OR teenager*[tiab] OR youth*[tiab] OR youngster*[tiab] | **3021991** |
| **#7** | "Feasibility Studies"[Mesh] OR "Evaluation Studies" [Publication Type] OR implementation[tiab] OR evaluation[tiab] OR fidelity[tiab] OR adherence[tiab] OR dose[tiab] OR delivery[tiab] OR responsiveness[tiab] OR differentiation[tiab] OR quality[tiab] OR process[tiab] OR dosage[tiab] OR completeness[tiab] OR compliance[tiab] OR adaptation[tiab] OR feasibility[tiab] OR satisfaction[tiab] | **4825100** |
| **#6** | "Health Promotion"[Mesh] OR health promotion[tiab] OR program*[tiab] OR intervention*[tiab] | **1446669** |
| **#5** | #1 OR #2 OR #3 OR #4 | **1750463** |
| **#4** | "Sedentary Lifestyle"[Mesh] OR sedentary[tiab] OR sitting[tiab] OR "TV time"[tiab] OR "TV viewing"[tiab] OR "watching TV"[tiab] OR "computer time"[tiab] OR "computer use"[tiab] OR "screen time"[tiab] OR "screen-time"[tiab] OR "television"[tiab] OR "gaming" [tiab] OR physical inactivit*[tiab] | **64763** |
| **#3** | “Exercise” [Mesh] OR “physical fitness” [Mesh] OR "Physical Education and Training" [Mesh] OR "Sports" [Mesh] OR exercis*[tiab] OR physical education[tiab] OR physical activit*[tiab] OR sport*[tiab] | **473783** |
| **#2** | "Food"[Mesh] OR "Eating"[Mesh] OR “Fruit"[Mesh] OR "Vegetables"[Mesh] OR eating[tiab] OR drinking[tiab] OR food[tiab] OR nutrition[tiab] OR fruit*[tiab] OR vegetable*[tiab] | **1083934** |
| **#1** | "Overweight"[Mesh] OR overweight*[tiab] OR obes*[tiab] | **296143** |

**Embase search strategy 2017 October 3^rd^**

| **#** | **Query** | **Results** |
| --- | --- | --- |
| **#11** | #10 AND [2001-2017]/py | **5374** |
| **#10** | #5 AND #6 AND #7 AND #8 AND #9 | **5873** |
| **#9** | 'school'/exp OR school*:ab,ti OR teacher*:ab,ti | **549415** |
| **#8** | 'adolescent'/de OR 'school child'/exp OR 'preschool child'/exp OR adolescen*:ab,ti OR child*:ab,ti OR teen:ab,ti OR teens:ab,ti OR teenager*:ab,ti OR youth*:ab,ti OR youngster*:ab,ti | **2861884** |
| **#7** | 'feasibility study'/exp OR 'evaluation study'/exp OR implementation:ab,ti OR evaluation:ab,ti OR fidelity:ab,ti OR adherence:ab,ti OR dose:ab,ti OR delivery:ab,ti OR responsiveness:ab,ti OR differentiation:ab,ti OR quality:ab,ti OR process:ab,ti OR dosage:ab,ti OR completeness:ab,ti OR compliance:ab,ti OR adaptation:ab,ti OR feasibility:ab,ti OR satisfaction:ab,ti | **6152425** |
| **#6** | 'health promotion'/exp OR 'health program'/exp OR health AND promotion:ab,ti OR program*:ab,ti OR intervention*:ab,ti | **1849456** |
| **#5** | #1 OR #2 OR #3 OR #4 | **2330721** |
| **#4** | 'sedentary lifestyle'/exp OR 'sitting'/exp OR 'television viewing'/exp OR 'physical inactivity'/exp OR sedentary:ab,ti OR sitting:ab,ti OR 'tv time':ab,ti OR 'tv viewing':ab,ti OR 'watching tv':ab,ti OR 'computer time':ab,ti OR 'computer use':ab,ti OR 'screen time':ab,ti OR 'screen-time':ab,ti OR 'television':ab,ti OR 'gaming':ab,ti OR (physical NEXT/1 inactivit*):ab,ti | **89177** |
| **#3** | 'exercise'/exp OR 'fitness'/exp OR 'physical education'/exp OR 'sport'/exp AND 'physical activity'/exp OR exercis*:ab,ti OR 'physical education':ab,ti OR (physical NEXT/1 activit*):ab,ti OR sport*:ab,ti | **511944** |
| **#2** | 'food'/exp OR 'eating'/exp OR 'fruit'/exp OR 'vegetable'/exp OR eating:ab,ti OR food:ab,ti OR nutrition:ab,ti OR fruit*:ab,ti OR vegetable*:ab,ti OR 'drinking'/exp OR drinking:ab,ti OR 'food intake'/de | **1465175** |
| **#1** | 'obesity'/exp OR overweight*:ab,ti OR obes*:ab,ti | **494156** |

**CINAHL search strategy 2017 October 3^rd^**

| **#** | **Query** | **Results** |
| --- | --- | --- |
| **S13** | S5 AND S6 AND S7 AND S10 AND S11  Limiters - Published Date: 20010101-20181231 | **1295** |
| **S12** | S5 AND S6 AND S7 AND S10 AND S11 | **1440** |
| **S11** | (MH "Schools") OR (MH "Schools, Elementary") OR (MH "Schools, Middle") OR (MH "Schools, Secondary") OR (MH "Teachers") OR TI (school* OR teacher*) OR AB (school* OR teacher*) | **42569** |
| **S10** | S8 OR S9 | **499554** |
| **S9** | TI (adolescen* OR child* OR teen OR teens OR teenager* OR youth* OR youngster*) OR AB (adolescen* OR child* OR teen OR teens OR teenager* OR youth* OR youngster*) | **281489** |
| **S8** | *Limiters - Age Groups: Child, Preschool: 2-5 years, Child: 6-12 years, Adolescent: 13-18 years* | **414747** |
| **S7** | (MH "Evaluation") OR (MH "Program Evaluation") OR TI (implementation OR evaluation OR fidelity OR adherence OR dose OR delivery OR responsiveness OR differentiation OR quality OR process OR dosage OR completeness OR compliance OR adaptation OR feasibility OR satisfaction) OR AB (implementation OR evaluation OR fidelity OR adherence OR dose OR delivery OR responsiveness OR differentiation OR quality OR process OR dosage OR completeness OR compliance OR adaptation OR feasibility OR satisfaction) | **583838** |
| **S6** | (MH "Health Promotion") OR DE "School Based Intervention" OR TI (“health promotion” OR program* OR intervention*) OR AB (“health promotion” OR program* OR intervention*) | **390623** |
| **S5** | S1 OR S2 OR S3 OR S4 | **346386** |
| **S4** | (MH "Life Style, Sedentary") OR TI (sedentary OR sitting OR "TV time" OR "TV viewing" OR "watching TV" OR "computer time" OR "computer use" OR "screen time" OR "screen-time" OR "television" OR "gaming" OR “physical inactivit*”) OR AB (sedentary OR sitting OR "TV time" OR "TV viewing" OR "watching TV" OR "computer time" OR "computer use" OR "screen time" OR "screen-time" OR "television" OR "gaming" OR “physical inactivit*”) | **16232** |
| **S3** | (MH "Exercise+") OR (MH "Physical Fitness") OR (MH "Physical Education and Training+") OR (MH "Sports+") OR TI (exercis* OR “physical education” OR “physical activit*” OR sport*) OR AB (exercis* OR “physical education” OR “physical activit*” OR sport*) | **160182** |
| **S2** | (MH "Food+") OR (MH "Eating") OR (MH "Eating Behavior") OR (MH "Fruit+") OR (MH "Vegetables+") OR TI (eating OR food OR nutrition OR fruit* OR vegetable*) OR AB (eating OR food OR nutrition OR fruit* OR vegetable*) | **155765** |
| **S1** | MH "Obesity" OR MH "Obesity, Morbid" OR TI (overweight* OR obes*) OR AB (overweight* OR obes*) | **59002** |

**COCHRANE search strategy 2017 October 3^rd^**

| **#** | **Query** | **Results** |
| --- | --- | --- |
| **S11** | #10 (Publication Year from 2001 to 2017) | **1169** |
| **S10** | #5 AND #6 AND #7 AND #8 AND #9 | **1852** |
| **S9** | school*:ti,ab,kw OR teacher*:ti,ab,kw | **22979** |
| **S8** | adolescen*:ti,ab,kw OR child*:ti,ab,kw OR teen:ti,ab,kw OR teens:ti,ab,kw OR teenager*:ti,ab,kw OR youth*:ti,ab,kw OR youngster*:ti,ab,kw | **183594** |
| **S7** | implementation:ti,ab,kw OR evaluation:ti,ab,kw OR fidelity:ti,ab,kw OR adherence:ti,ab,kw OR dose:ti,ab,kw OR delivery:ti,ab,kw OR responsiveness:ti,ab,kw OR differentiation:ti,ab,kw OR quality:ti,ab,kw OR process:ti,ab,kw OR dosage:ti,ab,kw OR completeness:ti,ab,kw OR compliance:ti,ab,kw OR adaptation:ti,ab,kw OR feasibility:ti,ab,kw OR satisfaction:ti,ab,kw | **444738** |
| **S6** | (health:ti,ab,kw AND promotion:ti,ab,kw) OR program*:ti,ab,kw OR intervention*:ti,ab,kw | **194686** |
| **S5** | #1 OR #2 OR #3 OR #4 | **149846** |
| **S4** | sedentary:ti,ab,kw OR sitting:ti,ab,kw OR 'tv time':ti,ab,kw OR 'tv viewing':ti,ab,kw OR 'watching tv':ti,ab,kw OR 'computer time':ti,ab,kw OR 'computer use':ti,ab,kw OR 'screen time':ti,ab,kw OR 'screen-time':ti,ab,kw OR 'television':ti,ab,kw OR 'gaming':ti,ab,kw OR “physical inactivit*”:ti,ab,kw | **26654** |
| **S3** | exercis*:ti,ab,kw OR 'physical education':ti,ab,kw OR “physical activit*”:ti,ab,kw OR sport*:ti,ab,kw | **72253** |
| **S2** | eating:ti,ab,kw OR food:ti,ab,kw OR nutrition:ti,ab,kw OR fruit*:ti,ab,kw OR vegetable*:ti,ab,kw OR drinking:ti,ab,kw | **49863** |
| **S1** | overweight*:ti,ab,kw OR obes*:ti,ab,kw | **25532** |

**PsycINFO search strategy 2017 October 3^rd^**

| **#** | **Query** | **Results** |
| --- | --- | --- |
| **S13** | S5 AND S6 AND S7 AND S10 AND S11  Limiters - Date Published: 20010101-20181231 | **1916** |
| **S12** | S5 AND S6 AND S7 AND S10 AND S11 | **2667** |
| **S11** | (DE "Schools" OR DE "Elementary Schools" OR DE "High Schools" OR DE "Junior High Schools" OR DE "Middle Schools") OR (DE "Teachers" OR DE "Elementary School Teachers" OR DE "High School Teachers" OR DE "Junior High School Teachers" OR DE "Middle School Teachers") OR TI (school* OR teacher*) OR AB (school* OR teacher*) | **428206** |
| **S10** | S8 OR S9 | **979767** |
| **S9** | TI (adolescen* OR child* OR teen OR teens OR teenager* OR youth* OR youngster*) OR AB (adolescen* OR child* OR teen OR teens OR teenager* OR youth* OR youngster*) | **780481** |
| **S8** | Limiters - Age Groups: Preschool Age (2-5 yrs), School Age (6-12 yrs), Adolescence (13-17 yrs) | **588968** |
| **S7** | DE "Program Evaluation" OR DE "Evaluation" OR DE "Educational Program Evaluation" OR TI (implementation OR evaluation OR fidelity OR adherence OR dose OR delivery OR responsiveness OR differentiation OR quality OR process OR dosage OR completeness OR compliance OR adaptation OR feasibility OR satisfaction) OR AB (implementation OR evaluation OR fidelity OR adherence OR dose OR delivery OR responsiveness OR differentiation OR quality OR process OR dosage OR completeness OR compliance OR adaptation OR feasibility OR satisfaction) | **1202925** |
| **S6** | DE "Health Promotion" OR DE "School Based Intervention" OR TI (“health promotion” OR program* OR intervention*) OR AB (“health promotion” OR program* OR intervention*) | **606692** |
| **S5** | S1 OR S2 OR S3 OR S4 | **274469** |
| **S4** | DE "Television Viewing" OR TI (sedentary OR sitting OR "TV time" OR "TV viewing" OR "watching TV" OR "computer time" OR "computer use" OR "screen time" OR "screen-time" OR "television" OR "gaming" OR “physical inactivit*”) OR AB (sedentary OR sitting OR "TV time" OR "TV viewing" OR "watching TV" OR "computer time" OR "computer use" OR "screen time" OR "screen-time" OR "television" OR "gaming" OR “physical inactivit*”) | **30740** |
| **S3** | DE "Exercise" OR DE "Physical Activity" OR DE "Physical Fitness" OR DE "Physical Education" OR DE "Sports" OR TI (exercis* OR “physical education” OR “physical activit*” OR sport*) OR AB (exercis* OR “physical education” OR “physical activit*” OR sport*) | **115247** |
| **S2** | DE "Eating Behavior" OR DE "Food" OR DE "Food Intake" OR TI (eating OR food OR nutrition OR fruit* OR vegetable*) OR AB (eating OR food OR nutrition OR fruit* OR vegetable*) | **129356** |
| **S1** | DE "Overweight" OR DE "Obesity" OR TI (overweight* OR obes*) OR AB (overweight* OR obes*) | **40008** |

**ERIC search strategy 2017 October 3^rd^**

| **#** | **Query** | **Results** |
| --- | --- | --- |
| **S13** | S5 AND S6 AND S7 AND S10 AND S11  Limiters - Date Published: 20010101-20181231 | **1146** |
| **S12** | S5 AND S6 AND S7 AND S10 AND S11 | **2845** |
| **S11** | (DE "Schools" OR DE "Elementary Schools" OR DE "Secondary Schools" OR DE "Junior High Schools" OR DE "Middle Schools" OR DE "High Schools") OR (DE "Teachers" OR DE "Elementary School Teachers" OR DE "Middle School Teachers" OR DE "Secondary School Teachers") OR TI (school* OR teacher*) OR AB (school* OR teacher*) | **677503** |
| **S10** | S8 OR S9 | **346619** |
| **S9** | TI (adolescen* OR child* OR teen OR teens OR teenager* OR youth* OR youngster*) OR AB (adolescen* OR child* OR teen OR teens OR teenager* OR youth* OR youngster*) | **334727** |
| **S8** | (DE "Adolescents" OR DE "Early Adolescents" OR DE "Late Adolescents") OR (DE "Children" OR DE "Young Children") | **103515** |
| **S7** | (DE "Program Evaluation") OR (DE "Evaluation") OR TI (implementation OR evaluation OR fidelity OR adherence OR dose OR delivery OR responsiveness OR differentiation OR quality OR process OR dosage OR completeness OR compliance OR adaptation OR feasibility OR satisfaction) OR AB (implementation OR evaluation OR fidelity OR adherence OR dose OR delivery OR responsiveness OR differentiation OR quality OR process OR dosage OR completeness OR compliance OR adaptation OR feasibility OR satisfaction) | **471796** |
| **S6** | DE "Health Promotion" OR TI (“health promotion” OR program* OR intervention*) OR AB (“health promotion” OR program* OR intervention*) | **443024** |
| **S5** | S1 OR S2 OR S3 OR S4 | **102488** |
| **S4** | (DE "Television Viewing") OR (DE "Computer Use") OR TI (sedentary OR sitting OR "TV time" OR "TV viewing" OR "watching TV" OR "computer time" OR "computer use" OR "screen time" OR "screen-time" OR "television" OR "gaming" OR “physical inactivit*”) OR AB (sedentary OR sitting OR "TV time" OR "TV viewing" OR "watching TV" OR "computer time" OR "computer use" OR "screen time" OR "screen-time" OR "television" OR "gaming" OR “physical inactivit*”) | **24496** |
| **S3** | (DE "Physical Activities" OR DE "Athletics" OR DE "Dance" OR DE "Exercise" OR DE "Aquatic Sports" OR DE "College Athletics" OR DE "Racquet Sports" OR DE "Team Sports" OR DE "Track and Field" OR DE "Womens Athletics") OR (DE "Physical Fitness" OR DE "Health Related Fitness") OR (DE "Physical Education" OR DE "Adapted Physical Education" OR DE "Movement Education") OR TI (exercis* OR “physical education” OR “physical activit*” OR sport*) OR AB (exercis* OR “physical education” OR “physical activit*” OR sport*) | **57003** |
| **S2** | DE "Food" OR DE "Nutrition" OR TI (eating OR food OR nutrition OR fruit* OR vegetable*) OR AB (eating OR food OR nutrition OR fruit* OR vegetable*) | **24979** |
| **S1** | DE "Obesity" OR TI (overweight* OR obes*) OR AB (overweight* OR obes*) | **3123** |
