# Supplementary material for: Measuring implementation fidelity of school-based obesity prevention programmes: a systematic review
Source: Int J Behav Nutr Phys Act. 2018 Aug 13;15:75. doi: 10.1186/s12966-018-0709-x (PMC6088402; doi:10.1186/s12966-018-0709-x)
Supplement: Supplementary file 3 — Data extraction. Full overview of the data extraction of the included studies. (DOCX 84 kb) [file 12966_2018_709_MOESM3_ESM.docx]

**Table 3. Data extraction**

| **Study** | **Programme** | | | **Process evaluation** | | | | | |
| --- | --- | --- | --- | --- | --- | --- | --- | --- | --- |
| **Author & year of publication**  **Programme**  **Country of delivery** | **Programme characteristics** | **Setting**  **Target group**  **Implementer** | **Theoretical framework** | **Evaluated fidelity components** | **Definition fidelity component** | **Data collection**  **Method and timing** | **Subject of evaluation** | **Summary of the results of the fidelity component** | **Relation between fidelity component and programme outcomes** |
| Alaimo (2015) (78)  Project FIT  United States | Programme consisting of school, community and social marketing elements aimed to improve DB and increase PA  Duration 6 weeks | Primary schools (3^rd^-5^th^ grade) (n=4 in intervention arm)  Age not reported (n=320 in intervention arm)  Teachers (n=129) | No theory reported | Dose | Hours spent per classroom per year on nutrition education; Number of students participating in taste testing | Sign-in sheets and standardised forms, monthly | Teacher | Teachers provided on average 4.7h classroom education in year 1 and 6.5h in year 2. 3011 students attended tasting sessions in the classroom and over 1500 in the cafeteria | Not reported |
| Almas (2013) (76)  School based PA programme  Pakistan | Programme consisting of 4 times per week 30 min PA aimed to reduce blood pressure and BMI  Duration 20 weeks | Primary schools (n=2 in intervention arm)  Girls aged 9-11 years (n=131 intervention arm)  Physical trainers (n=not reported) | No theory reported | Dose | Fidelity: the proportion of planned PA sessions actually held | Logbook maintained by researchers during the programme | Teacher | Out of the total 80 planned sessions, 69 (86%) were successfully held | Not reported |
|  |  |  |  | Responsiveness | Acceptability to intervention | Student questionnaire after programme completion | Student | 92% of students enjoyed the PA sessions | Not reported |
| Aarestrup (2015) &  Jørgensen (2015, 2017) (51, 77, 87)  Boost study  Denmark | Programme consisting of daily provision of free FV, a pleasant eating environment, classroom curricular activities, and parental newsletters  aimed to increase FV consumption  Duration 9 months | Secondary schools (n=20)  Students aged 13 years (n=1,121)  Teachers (n=114) | Key process evaluation components defined by Steckler and Linnan | Adherence | Fidelity: extent to which the intervention components were delivered by teachers according to the teacher manual | Teacher questionnaire midway and after programme completion | Teacher | Daily FV provision fidelity ranged from 50-94% | Not reported |
|  |  |  |  | Dose | Dose delivered: the proportion of the components which was delivered | Teacher questionnaire midway and after programme completion | Teacher | Classroom curriculum delivery ranged from 0-10.7 activities. In 11 schools, 4 of the 6-parental newsletters were uploaded. Daily provision of FV decreased over time (85 to 64%) | Not reported |
|  |  |  |  |  | Dose received: the extent to which pupils received the components | Student questionnaire 3 times during the programme | Student | Classroom curriculum received ranged from 3.5-11.4 activities. 68% had time allocated to eat FV | Dose received was associated with an increase in students' knowledge of recommendation for vegetable intake and not associated with an increase in knowledge of recommendation for fruit intake, taste preferences or situational norms |
|  |  |  |  | Responsiveness | Students' and teachers’ appreciation of the Boost intervention | Teacher questionnaire midway and after programme completion | Teacher | Students having a good time 83% | Not reported |
|  |  |  |  |  |  | Student questionnaire 3 times during the programme | Student | Students having a good time 54% | Not reported |
| Barr-Anderson (2012) (75)  Presidential Active Lifestyle Award (PALA)  United States | Programme consisting of classroom, peer-led and home component  aimed to promote children to participate in at least 60 min of daily PA  Duration 6 weeks | Primary schools (n=2 in intervention arm)  Students aged 8 years and higher (n=87 in intervention arm)  Teachers (n=4) | No theory reported | Dose | Programme fidelity: percent completion of activities for each session | Classroom observations at least once by each of the 6 sessions and at each of the 4 classroom teachers | Teacher | 100% completion of programme components in the classroom | Not reported |
|  |  |  |  | Quality of delivery | Programme fidelity: quality of activity facilitation by the peer leaders | Classroom observation at least once by each of the 6 sessions and at each of the 4 classroom teachers | Teacher | The quality of the peer leader’s ability to facilitate activities was rated on average 4.2 (scale 1=ineffective, 5=excellent) | Not reported |
|  |  |  |  | Responsiveness | Programme receptivity and satisfaction | Teacher questionnaire after programme completion | Teacher | Teacher liked the peer leadership component, but found the programme too short | Not reported |
|  |  |  |  |  |  | Classroom observation at least once by each of the 6 sessions at each of the 4 classroom, teachers and student questionnaire after programme completion | Student | Average rating for the student/teacher response to the session was 4.4 (scale 1=not enthusiastic, 5=extremely enthusiastic). Students reported that the DVD was their favourite component (60%) | Not reported |
| Battjes-Fries (2016) (79)  Taste Lessons  Netherlands | Programme consisting of standard lessons and optional lessons  aimed to increase interest in food, and knowledge and skills regarding making HE choices  Duration 2 school years | Primary schools (grade 1-8) (n=12 schools)  Students aged 8-12 years (n=392)  Teachers (n=20 teachers) | No theory reported | Dose | Indication of standard activities per lesson that were delivered | Teacher checklist at each lesson | Teacher | Teachers implemented on average 4.6 lessons and 28% of the activities | Positive association between dose and students' change in subjective norm of teachers on short term |
|  |  |  |  | Responsiveness | Appreciation: extent to which the teachers appreciated the lessons, how much they liked the lessons and how feasible to implement | Teacher questionnaire one month after the intervention | Teacher | Teachers rated the programme on average 7.9 (10-point scale) and perceived the lessons nice and feasible to implement | Teacher appreciation showed inverse association with attitude and students' subjective norm of the teachers on short term |
|  |  |  |  |  | Appreciation: extent to which they liked the lessons | Student questionnaire one month after the intervention | Student | Children rated the programme on average 7.8 (10-point scale) and were most positive about practical activities | Student appreciation was significantly associated with awareness, emotion and student subjective norm of teacher on short term, and with attitude and students subjective norm of parents on the long term |
| Bere (2005) (74)  Fruit and Vegetables Make the Marks (FVMM)  Norway | Programme consisting of classroom, parental and school food programme aimed to increase overall FV consumption  Duration 7 months | Primary schools (n=8 in intervention arm)  Students aged 11-12 years (n=190 in intervention arm)  Teachers (n=8) | No theory reported | Dose | Implementation | Teacher questionnaire twice after programme completion | Teacher | Implementation ranged from 2.5 to 7 of the sessions (scale: 0=not implemented, 7=fully implemented)  Mean score of the newsletter handout was 1.5 of the 6 | No relation between the amount of the curriculum implemented and FV intake. Students with high usage of newsletter showed significant higher intake of FV compared to low usage of newsletters |
|  |  |  |  | Responsiveness | Pupils enjoyment | Student questionnaire twice after programme completion | Student | The mean student enjoyment was 3.3 (scale: 5=low, 8=high) | Students who indicated high enjoyment showed significant higher intake of FV compared with the students who indicated low enjoyment |
| Bergh (2012) (73)  The Health in Adolescent study (HEIA)  Norway | Programme consisting of individual, group and environmental strategies and components aimed to promote healthy weight development  Duration 20 months | Primary schools (6^th^ and 7^th^ grade) (n=12 in intervention arm)  Students aged 11-13 years (n= 510 in intervention arm)  Teachers (n=not reported) | No theory reported | Dose | Dose received: degree of exposure to or participation in the PA and SB intervention components | Student questionnaire mid-way and after programme completion | Student | At mid-way 273 (56%) of the students in the intervention group reported a high intervention dose received, whereas 156 (31%) reported this at the time of post-programme | The effect on determinants (physiological and social-environmental) for PA and SB was influenced by the adolescents’ degree of exposure to and participation in the programme |
| Bessems (2011, 2013) (71, 72)  Krachtvoer  Netherlands | Programme consisting of classroom curriculum aimed to increase intake of fruit and fruit juice, decrease the consumption of high-fat snacks and increase breakfast frequency and quality  Duration 3 months | Secondary school (n=13 schools)  Students aged 12-14 years (n=1117)  Teachers (n=22) | No theory reported | Adherence | Adherence: implementation as intended by the developers | Teacher logbooks after each lesson and classroom observations, timing not reported | Teacher | The intended order of the 8 lessons was followed by all but one teacher. 10 of the 18 teachers implemented the programme over the intended period and deviations from the programme included changing working methods and using additional programme materials | Not reported |
|  |  |  |  | Dose | Completeness of implementation: the proportion of activities proposed by the programme that was actually used | Teacher logbook after each lesson | Teacher | On average teachers implemented 6.1 of the 7 fixed lessons and 4.6 of the 7 optional activities | Completeness of implementation related to an increase in fruit consumption in the short term and completeness of implementation of practical activities related to an increase in fruit consumption in the short and longer term |
|  |  |  |  | Responsiveness | Teacher appreciation | Teacher logbook after each lesson | Teacher | Teachers scored the fixed lessons 7.0, optional activities 7.5 and supportive materials 7.4 (10-point scale) | Not reported |
|  |  |  |  |  | Student appreciation | Student evaluation forms once after programme completion and random classroom observations | Student | Students rated the programme on average 7.3 out of 10. Students responded most enthusiastic to practical activities (tasting, recipe preparation) and least to planning activities | Not reported |
| Blaine (2017) (80)  MA-CORD project  United States | Programme consisting of curriculum delivery, provision of PA supplies and educational materials  aimed to prevent obesity or control obesity  Duration 2 years | Primary schools (n= 2 communities: n=6 schools in community 1; n=22 schools in community 2)  Students aged 2-12 years (n=not reported)  Teachers (n=124 in year 1; n=128 in year 2) | Taxonomy of outcomes for implementation research by Proctor | Dose | Implementation fidelity: proportion of MA-CORD lessons taught | Teachers questionnaire at the end of each year | Teacher | Community 1 teachers delivered 5.8 of the 6 lessons in year 1 and dropped slightly in year 2; Community 2 teachers delivered 3.6 of the 6 lessons in year 1, but increased in year 2 | Not reported |
|  |  |  |  | Responsiveness | Appropriateness: lessons perceived as positive addition to the curriculum | Teachers questionnaire at the end of each year | Teacher | All teachers (100%) agreed that the lessons were a positive addition to the curriculum | Not reported |
| Blom-Hoffman (2004) (69)  Five A Day for Better Health  United States | Programme consisting of a classroom, lunchtime, and home component aimed to increase FV knowledge and vegetable consumption during school lunch  Duration not reported | Primary Schools (n=6 classes)  Students aged 4-7 years (n=150)  Teachers and classroom assistants (n=not reported) | No theory reported | Adherence | Treatment integrity: lessons implemented as intended | Observations using a checklist during 28% of the lessons and 21% of the lunches | Teacher | Treatment integrity for the knowledge-based classroom component was high, for the behaviourally based component lower than the knowledge-based component and for lunchtime extremely variable | Not reported |
|  |  |  |  | Responsiveness | Acceptability: teacher and classroom satisfaction | Teacher questionnaire once in the last week of the programme | Teacher | All teachers indicated that the programme was socially valid | Not reported |
|  |  |  |  |  | Acceptability: student satisfaction | Student questionnaire once in the last week of the programme | Student | On average acceptability was reported high | Not reported |
| Blom-Hoffman (2008) (70)  Athletes in Service FV promotion programme  United States | Programme consisting of whole school, classroom, lunchroom, and family activities  aimed to increase children’s nutrition knowledge, preference for FV, actual eating behaviours during school lunch, availability and accessibility of FV in the home and children’s weight status  Duration winter 2006 – spring 2008 | Primary schools (n=2 in the intervention arm)  Students aged 5-9 years (not reported)  Teachers (n=15) and lunch aids (n=12) | No theory reported | Adherence | Implementation integrity: programme delivery compliance | Unannounced observations on 24% of programme implementation days and athlete’s daily logs | Teacher | Integrity for lunchtime procedures was high (range 75%- 100%). Morning announcements occurred on most school days (91% of monitoring days) | Not reported |
|  |  |  |  | Dose | Programme delivery | Teacher checklist on CD-ROM usage, timing not reported | Student | On average, students were exposed to 3 songs, 6 FV characters and 3 cooking videos | Not reported |
|  |  |  |  | Responsiveness | Teacher acceptability | Teacher questionnaire each classroom session | Teacher | Teachers were enthusiastic about the programme (mean 5.51 on 1-6 Likert scale) | Not reported |
|  |  |  |  |  | Implementation integrity: child engagement | Teacher questionnaire each classroom session | Student | Students paid very good attention to the CD-ROM (range 1.67-1.89, 3-point scale 0-2) and students seemed to enjoy using the CD-ROM (range 1.43-2.0, 3-point scale 0-2) | Not reported |
|  |  |  |  |  | Lunch aid acceptability | Lunch aid questionnaire, timing not reported | Teacher | Lunch aids found the programme highly acceptable (mean 5.62 on 1-6 Likert scale) | Not reported |
| Burgermaster (2017) (81)  Food, Health & Choices  United States | Programme consisting of class-room nutrition curriculum or wellness policy or both aimed at prevention of obesity  Duration 1 school year | Primary schools (n=10 curriculum arm; n=10 wellness arm)  Students mean age=10.6 (*n*=1140 curriculum; n=954 wellness)  Instructors (n=not reported) | No theory reported | Adherence | Percent completion | Instructor feedback form after each session | Teacher | Curriculum schools had a mean fidelity score of 95.2; Wellness schools 66.7 (max=100) | Fidelity was negatively related to energy-balance related outcomes |
|  |  |  |  | Quality of delivery | Teacher interest: teacher participation and teacher attitude | Instructor feedback form after each session | Teacher | Curriculum schools had a mean teacher interest score of 2.4; Wellness schools 2.5 (max=3) | Teacher interest was not significantly associated with any energy related outcomes |
|  |  |  |  | Responsiveness | Student satisfaction | Student questionnaire during last session | Student | Curriculum school students rated their overall satisfaction as 4.0 and wellness school students as 4.1 (max=5) | Student satisfaction was related to improves energy-balance related outcomes |
| Campbell (2015) (68)  Active for Life Year 5 (AFLY5)  United Kingdom | Programme consisting of lessons, homework, hand out for parents and school newsletters aimed to increase children’s PA and FV intake and decrease SB  Duration 11 months | Primary schools (n=30 in intervention arm)  Students aged 9-10 (n=not reported)  Teachers (n=44) and support staff | No theory reported | Adherence | Implementation fidelity: Lesson amendments | Observations and teacher logs after each lesson | Teacher | 28% of the lessons were amended. 89% of teachers amended the source or lesson content at least on 1 occasion | Not reported |
|  |  |  |  | Dose | Implementation fidelity: Number of lessons taught | Teacher logs after each lesson | Teacher | The mean number of lessons taught was 12.3 (77%). Delivery declined over time. The mean number of homework that was delivered was 6.2 (62%) | Not reported |
| Christian (2012) (67)  Project Tomato  United Kingdom | Programme consisting of classroom lessons and parental activities  aimed to improve children’s knowledge and awareness of dietary FV consumption  Duration 10 months | Primary Schools (24 in intervention arm)  Students aged 8-9 (n=311)  Teachers (n=19) | No theory reported | Dose | Implementation | Teacher questionnaire at different periods during the programme | Teacher | Implementation of the programme was low, 21% of school items were implemented | Total implementation of all school items showed no significant difference between programme implementation levels and follow-up FV intake |
|  |  |  |  | Responsiveness | Appreciation | Teacher questionnaire at different periods during the programme | Teacher | Teacher's mean appreciation for all school intervention items was 65.7% | Not reported |
|  |  |  |  |  |  | Student questionnaire at different periods during the programme | Student | Children's appreciation was high, with a mean of 73% | Appreciation was not significantly associated with change in children's FV intake |
| Dalton (2014) (66)  Winning With Wellness Project (WWW)  United States | Programme consisting of nutrition services, health education, PE, school health counselling, healthy school environment, health promotion for staff and family and community involvement aimed at promotion of HE and PA  Duration 9 months | Primary Schools (n=not reported)  Students aged 11-12 (n=144)  Teachers (n=75) | No theory reported | Dose | Degree of teacher implementation | Teacher questionnaire during two occasions in the study period | Teacher | Level of programme implementation for specific WWW components ranged from 40% to 65.7% | Not reported |
|  |  |  |  | Responsiveness | Usefulness/ helpfulness of the programme and its various components | Teacher questionnaire during two occasions in the study period | Teacher | The majority of teachers (88.5%, n = 62) found the overall WWW programme helpful to some degree | Not reported |
| Davis (2000) (65)  Gimme 5 Fruit and Vegetables for Fun and Health  United States | Programme consisting of curriculum, newsletters to families, video tapes, and family activities  aimed at increase of FV intake  Duration 6 weeks | Primary schools (n=16)  Students aged 9-11 years (n=not reported)  Teachers (n=69) | No theory reported | Dose | Fidelity of implementation delivery | Observations at least once per teacher, teacher questionnaire after each lesson and structured interview after programme completion | Teacher | Observations showed that about half of the curriculum activities were completed each year. In the questionnaire teachers completed 90% of all curriculum activities each year. In interviews teachers completed 80% to 91% of the activities | Not reported |
| Davis (2003) & Steckler (2003) (21, 64)  The Pathways study  United States | Programme consisting of a classroom curriculum, PA, food service and family events aimed to reduce the prevalence of obesity by focusing on healthy environments as well as diet and PA  Duration 3 years | Primary schools (n=21 in intervention arm)  Students aged 7-11years (n=584 in intervention arm)  Teachers (n=250) | No theory reported | Dose | Dose delivered: Extent and fidelity | Teacher evaluation forms at each session, teacher interview at the end of the semester and food service observations of a food service at least twice each fall semester and 3 times each spring semester | Teacher | 93.9% of the pathways curriculum lessons successfully taught in the 3^rd^-5^th^ grade. PE was taught on average 93.3% of the available days. Food service implementation increased from 51% to 87% | Not reported |
|  |  |  |  |  | Dose received: Extent to which the students perceived exposure to the programme | Student questionnaire at the end of each school year | Student | Each year there was a significant difference between control students’ perceived exposure to the Pathways interventions | Not reported |
| Day (2008) (63) & Naylor (2016) (92)  Action Schools! BC - HE  Canada | Programme consisting of FV classroom intervention aimed to increase FV intake  Duration 12 weeks | Primary schools (n=5 in intervention arm)  Students aged 9-12 years (n=246)  Teachers (n=not reported) | Theory of diffusion of innovations by Rogers | Dose | Fidelity; classroom delivery | Teachers activity logs, weekly | Teacher | Teachers delivered 64% of the requested 2 HE activities per week. On average 42% of the teacher were using AS! BC HE in their classroom with 39% providing classroom HE activities weekly and 28% monthly | Not reported |
|  |  |  |  | Quality of delivery | Teacher self-efficacy | Self-reported 5-point scale after scale-up | Teacher | Teachers demonstrated moderately high self-efficacy (3.5 on a 5-point scale) | Not reported |
|  |  |  |  | Responsiveness | How teacher perceived the programme | Self-reported one item question after scale-up | Teacher | Teachers demonstrated relatively high positive perceptions of the innovation attributes | Not reported |
| De Meij (2013) (62)  JUMP-in  Netherlands | Programme consisting of a policy, environmental and individual component aimed to promote sports participation and daily PA  Duration 2 years | Primary schools (n=9)  Students aged 6-12 years (n=not reported)  Teachers (n=not reported) | Key process evaluation components defined by Steckler and Linnan | Dose | Dose delivered: how much of the intended intervention was delivered as planned | Teacher questionnaire and structured interviews after two school years and documentary analysis during the programme | Teacher | There was a large variation within and between schools and not all components of the programme were implemented as planned | Not reported |
| Dubuy (2014) (82)  Health Scores  Belgium | Programme consisting of kick off and closure session at football clubs and a school programme aimed at promotion of a HE and PA  Duration 4 month | Primary schools (n=20 schools)  Students aged 10-14 years (n=165 in intervention arm)  Teachers (n=not reported) | No theory reported | Dose | Reported what topics had been discussed | Student questionnaire at follow-up measures | Student | Themes most commonly discussed were breakfast and vegetable consumption | Not reported |
|  |  |  |  | Responsiveness | Appreciation | Student questionnaire at follow-up measures | Student | Students rated the programme 7.8 on a 10-scale | Not reported |
| Dunton (2009) (61)  Exercise Your Options (EYC)  United States | Programme consisting of nutrition and PA lesson aimed to acquire skills to make healthy choices in all aspects of lives  Duration 1 school year | Primary and secondary school (n=not reported)  Students aged 11-13 years (n=683)  Teachers (n=16) | RE-AIM framework by Glasgow | Adherence | Extent to which the programme was delivered as intended | Observations during lessons over a 2-week period | Teacher | 75% of the lessons were implemented in the recommended order. All of the teachers conveyed 81% to 100% of the lesson content | Not reported |
|  |  |  |  | Dose | Number of lessons that ware delivered as intended | Teacher questionnaire, timing not reported | Teacher | 86% of the teachers implemented all eight lessons | Not reported |
| Dunton (2014) (60)  Shaping up my choices (SMC)  United States | Programme consisting of a nutrition education curriculum  aimed to promote HE behaviour and attitudes  Duration 10 weeks | Primary school (n=22 in intervention arm)  Students aged 8-9 years (n=651 in intervention arm)  Teachers (n=28) | RE-AIM framework by Glasgow | Adherence | Implementation: extent to which the programme was delivered as intended | Teacher questionnaire after programme completion and classroom observations  during four times in each classroom | Teacher | 39% of the teachers taught all material as written in the guide; 22% did not change lessons and 72% of the teachers assigned the family homework activities. In 83% of the observations the lessons were presented in the suggested order. 37% of the teachers made changes to content, 70% were closely following the lesson plans and 75% of presented information completely accurate | Not reported |
|  |  |  |  | Dose | No definition | Teacher questionnaire after programme completion | Teacher | 100% of the teachers reported implementing all ten lessons | Not reported |
| Eather (2016) (83)  CrossFit Teens  Australia | Programme consisting of CrossFit PE lessons aimed at improving health-related fitness  Duration 8 weeks | Secondary school (n=1)  Students aged 15-16 years (n=45)  CrossFit instructors | No theory reported | Dose | Adherence | Observations at each lesson | Teacher | All (100%) of the lessons were delivered with an attendance rate of 94% | Not reported |
|  |  |  |  | Responsiveness | Satisfaction | Student questionnaire after programme completion | Student | Students rated the programme 4.2 to 4.6 on a 5-point scale | Not reported |
| Elinder (2012)(59)  Stockholm County Implementation Programme (SCIP-school)  Sweden | Programme consisting of forming a local health team to address health policy and practices, including a meeting with parents aimed to improve eating habits, PA, self-esteem and a healthy body weight  Duration 2 years | Primary and secondary schools (n=9)  Students aged 6–16 years (n=764)  Local health teams (n= 4-11 per school) | No theory reported | Adherence | Fidelity: the extent to which a school had implemented all components in the programme according to the logic model | Interviews with health teams guided by a checklist on one occasion | Teacher | Programme fidelity was perfect for all schools, except for one. 27 of 56 components in the programme (48%) were fully implemented after two years | Not reported |
| Ezendam (2013) (58)  FATaintPHAT  Netherlands | Programme consisting of a computer-tailored intervention aimed to prevent excessive weight gain by improving DB, PA and SB  Duration 8 weeks | Secondary schools (n=9 in intervention arm)  Students aged 12-13 years (n=458 in intervention arm)  Teachers (n=19) | Key process evaluation components defined by Steckler and Linnan | Dose | Use of the intervention: number of students who completed all intervention modules | Computer log data and student questionnaire after programme completion | Student | The programme was completed by 81% of the students | No associations found between use and behavioural outcomes |
|  |  |  |  | Responsiveness | Appreciation of the intervention | Computer questionnaire after programme completion | Student | The average grade on a scale from 1 to 10 the students assigned the programme was 7.1 | No associations found between appreciation and behavioural outcomes |
| Gibson (2008) (57)  Physical activity across the curriculum (PAAC)  United States | Programme consisting of a classroom-based activity segment, and integrating PA with academic content  aimed to reduce obesity by improving PA  Duration 3 years | Primary schools (n=14 in intervention arm)  Students aged 7-11 years (n=2505 in intervention arm)  Teachers(n=135) | Key process evaluation components defined by Steckler and Linnan and Concepts in process evaluations by Baranowski and Stables | Dose | Fidelity: extent to which the teachers delivered the programme as originally planned | Online teacher log every week and principal questionnaire at the end of the school year | Teacher | 73% incorporated PA into language arts and 22% in math. Teachers incorporated 47 min at beginning and 65 min at end per week of active lessons. Most schools had PE classes 2 to 3 days per week with the majority of the classes least 30 min | Not reported |
|  |  |  |  | Quality of delivery | Self-efficacy | Online teacher questionnaire at the end of school year | Teacher | Most teachers indicated high level of confidence to demonstrate and instruct students on how to become physically active | Not reported |
|  |  |  |  | Responsiveness | Student enjoyment | Observations, weekly | Student | The majority of lessons were perceived as enjoyable (57%) or very enjoyable (36%) and only 6% of the lessons were perceived as neutral | Not reported |
| Griffin (2017) (86)  The WAVES study  United Kingdom | Programme consisting of daily PA, cooking workshop and signposting to local PA opportunities aimed to increase  PA levels and improve DB | Primary schools (n=24)  Students aged 6-7 years (n=not reported)  Teachers/school staff/football club staff (n=not reported)  Duration 1 term | Key process evaluation components defined by Steckler and Linnan | Adherence | Adherence/ fidelity | Teacher daily logbook and observations at least once per class | Teacher | Median implementation score across schools was 56/75 | Not reported |
| Hankonen (2017) (84)  School-based multilevel intervention  Finland | Programme consisting of student sessions and a teacher workshop  aimed to increase total PA  Duration 6 sessions | Secondary schools (n=1)  Students aged 15 years (n=26 in intervention arm)  Teachers (n=8) | No theory reported | Responsiveness | Acceptability | Teacher questionnaire after each workshop | Teacher | Teachers reported an on average 90 out of 100 score | Not reported |
|  |  |  |  |  |  | Student questionnaire after programme completion | Student | Students reported high satisfaction with the intervention (mean=6.29; scale 1-7) | Not reported |
| Harris (1998) (85)  5 A Day for Better Health Project  United States | Programme consisting of a classroom, cafeteria, supermarket, home and community component aimed to promote FV intake  Duration not reported | Primary schools (n=3)  Students age not reported (n=772)  Teachers (n=28) | No theory reported | Adherence | Project implemented as planned | Teachers' activity logs, timing not reported | Teacher | 82% of the teachers implemented the programme and 41% of the classroom materials were used | Not reported |
| Hildebrand (2012) (54)  Food and Fun for Everyone  United States | Programme consisting of a classroom curriculum aimed to influence students’ nutrition behaviours  Duration 6 weeks | Primary schools (n=9)  Students aged 9-11 years (n=812)  Paraprofessionals (n=9) | No theory reported | Dose | Fidelity to the curriculum protocol | Paraprofessional questionnaire on one occasion | Teacher | All 6 lessons were taught, generally during the 6-week period, with each lesson lasting 30-45 min and occasionally activities were eliminated | Not reported |
| Jan (2009) (53)  Shape It UP  United States | Programme consisting of interactive workshop, activity book and family guide, posters, website and educational field days aimed to increase awareness about the importance of HE and exercise  Duration 1 year | Primary schools (n=49)  Students aged 7-11 years (n=6,421)  Trained volunteers (n=not reported) | No theory reported | Responsiveness | Student satisfaction | Student questionnaire after programme completion | Student | Students reported high levels of satisfaction with the programme. Overall 91.7% selected 1 of the 3 most positive response categories of the 6-point scale | Not reported |
| Janssen (2013) (52)  The PLAYgrounds programme (PlAYground)  Netherlands | Programme consisting of physical alterations of the playground aimed to increase and stimulate PA during recess  Duration 10 months | Primary schools (n=4)  Students aged 6-12 years (n=730)  Teachers (n=59) | RE-AIM framework by Glasgow | Dose | Implementation: extent to which participating schools implemented the different elements of the programme | Teacher questionnaire after 5 months (follow up 1) and after 10 months (follow-up 2) and observations on 3 occasions per school | School | 5 of 7 elements were implemented at follow up 1 and 3 of 5 elements at follow up 2 | Not reported |
|  |  |  |  | Responsiveness | Satisfaction on the different elements of the programme | Student questionnaire after 5 months and after 10 months | Student | 85% of the students stated that the amount of joy was higher and 91% of children felt that they were more stimulated to PA | Not reported |
| Jurg (2006) (50)  JUMP-in, kids in motion  Netherlands | Programme consisting of school sports activities, pupil follow-up system, Class Moves!, Choose your Card!, parental information services and Activity-week aimed to promote PA  Duration 2 years | Primary schools (n=4 in intervention arm)  Students aged 4-12 years (n=369 in intervention arm)  Teachers (n=46) | No theory reported | Dose | Use of different programme components | Teacher questionnaire, structured in-depth interviews among PE teachers and participation lists, all at one occasion | Teacher | The level of use of programme components varied from 2-6 components | Not reported |
| King (2014) &  Lederer (2015) (47, 49)  HEROES  United States | Programme consisting of financial and technical support for data-based school-level interventions  aimed to increase PA and HE  Duration 3 years | Primary and secondary schools (n=17)  Students aged 6-19 years (n=6884)  School wellness coordinator (n=1 per school) | No theory reported | Adherence | Implementation fidelity: if the programme was  carried out as planned | Two site visits to conduct stakeholder interviews with the school wellness coordinator, school administrator and cafeteria manager and observations of school environment and materials | School | Schools’ total implementation scores ranged from 71.6% to 96.4% with a mean of 84.19% (SD = 7.58%). | Not reported |
| Lane (2017) (88)  Kids SIPsmartER  United States | Programme consisting of classroom lessons  aimed at reduction of sugar-sweetened beverages  Duration 6 lessons | Primary school (n=1)  Students mean age 11.7 years (n=43 in intervention arm)  Teachers (n=8) | No theory reported | Responsiveness | Acceptability | Teacher questionnaire after each lesson | Teacher | Teachers found the programme acceptable | Not reported |
|  |  |  |  |  |  | Student questionnaire at first follow-up | Student | Students liked the lessons | Not reported |
| Larsen (2015) (48)  Nutrition Pathfinders  United States | Programme consisting of class-room lessons  aimed to improve nutrition knowledge  Duration 4-10 weeks | Primary Schools (n=27 in intervention arm)  Students aged 9-10 years (n=971 in intervention arm)  Teachers (n=27) | RE-AIM framework by Glasgow | Adherence | Degree to which the teachers followed the teacher’s guide | Classroom observations on 1-3 occasions and teacher questionnaire after programme completion | Teacher | High percent of teachers followed the teacher guide. 17 out of 24 teachers presented most of the material written in the teacher’s guide and all teachers presented at least half of it | Not reported |
|  |  |  |  | Dose | Percentage of teachers completing the lessons | Classroom observations by on 1-3 occasions and teacher questionnaire after programme completion | Teacher | Teachers completed 80% of the materials. 88.9% completed all lessons, with a duration between 54 and 58 min per lesson | Not reported |
|  |  |  |  | Responsiveness | Extent to which students were cooperative and participated during the lessons | Teacher questionnaire after programme completion | Student | Students were either very or somewhat cooperative and well-behaved during the lessons | Not reported |
| Lee (2013) &  Gray (2015) (46, 56)  Choice control & change  United states | Programme consisting of education curriculum aimed to improve EBRBs  Duration 8-10 weeks | Primary and secondary schools (n=5 schools in intervention arm)  Students aged 11-13 years (n=562 in intervention arm)  Teachers (n=8) | Probabilistic mechanistic model of programme delivery by Baranowski and Jago | Adherence | Teacher implementation: faithfulness to the curriculum | Classroom observations, weekly | Teacher | Mean of faithfulness to the curriculum was 76% (range 62-76%) | High teacher implementation positively impacted students sweetened beverages, packaged snacks, fast food behaviours and psychological indicators. Medium and high teacher implementation showed significant improvement in walking, stair climbing and in reducing screen time |
|  |  |  |  | Dose | Teacher implementation: Lesson completion | Self-reported checklist form that was specific to each lesson, weekly during meetings with teachers and coordinators | Teacher | Mean of lesson completion rate was 70% (range 60-93%) |  |
|  |  |  |  | Responsiveness | Student reception:  extent to which students actively engaged in the lesson activities | Classroom observations, weekly and student questionnaire after programme completion | Student | Mean student engagement was 72% (range 49-100%) and means student satisfaction was 2.9 | How well students were engaged in the curriculum activities influenced students EBRBs and psychosocial outcomes |
| Lehto (2014) (45)  PRO GREENS intervention  Belgium, Bulgaria, Finland, Germany, Greece, Iceland, The Netherlands Norway, Portugal, Slovenia, Sweden | Programme consisting of classroom sessions, FV snacks, bring a dish and taste tests  aimed to promote consumption of FV  Duration 8 months | Primary schools (n=19)  Students aged 11 years (n=1123)  Teachers (n=71) | No theory reported | Dose | Degree of implementation | Teacher questionnaire during the follow-up study (1 year) | Teacher | 42% of the teachers implemented at least 7 lessons, 33% of the teachers implemented FV snacks at least once a weak, 71% of the teachers implemented bring-a-dish and 67% of the teachers implemented taste tests | Both low and high degree of implementation predicted an increase in fruit intake via a change in the knowledge of recommendations, but a high degree of implementation showed a stronger effect |
| Levine (2002) (24)  Team Nutrition  United States | Programme consisting of interactive classroom lessons and wider school community activities  aimed to change children’s eating behaviours  Duration not reported | Primary Schools (n=7 school districts, including 19 schools)  Students aged 4- 10 years (n=almost 300 classrooms)  Teachers (n=around 200) | No theory reported | Adherence | Fidelity | Teacher observations during the programme | Teacher | Teacher used the lessons materials about two thirds of the recommended time | Not reported |
|  |  |  |  | Dose | Implementation | Teacher activity logs during the programme | Teacher | On average teachers taught more than 7 of the 8 or 9 lessons from each module with an average duration of 2 hours. Nutrition students were exposed to an average of 14.4 hours of lessons in phase 1 and 16 hours in phase 2 | Not reported |
| Little (2015) (89)  Pathways programme  United States | Programme consisting of classroom lessons  aimed at SB, junk food intake, FV intake and PA  Duration 7 weeks in grade 4 and 5 weeks in grade 5 | Primary school (n=12)  Students age not reported (n=581)  Teachers (n=69) | No theory reported | Dose | Implementation fidelity | Teacher self-reported standard form after each lesson and observations, timing not reported | Teacher | 81.1% of teachers implemented 15 lessons in grade 4 and 97.6% of teachers implemented 10 lessons in grade 5 | Implementation fidelity was associated with higher intentions to eat healthfully, but not with actual behaviours |
|  |  |  |  | Quality of delivery | Quality of delivery: teacher's enthusiasm, integrity and quality | Teacher self-reported standard form after each lesson and observations, timing not reported | Teacher | Mean quality score ranged from 3.6-4.9 in the 4th and 5^th^ grade (range 1-5) | Not reported |
|  |  |  |  | Responsiveness | Teacher beliefs | Teacher self-reported standard form after each lesson | Teacher | Mean teacher beliefs scores ranged from 3.2-4.06 in the 4^th^ and 5^th^ grade (range 1-5) | Not reported |
|  |  |  |  |  | Participant engagement | Teacher self-reported standard form after each lesson | Student | Mean participant engagement ranged from 3.4-4.6 in the 4^th^ and 5^th^ grade (range 1-4) | Not reported |
| Lubans (2011) (23)  Physical Activity Leaders programme (PALs)  Australia | Programme consisting of school sport sessions, interactive seminars, lunch time activities, PA, nutrition handbooks, leadership sessions and pedometers aimed to encourage adolescent boys to become PA leaders  Duration 6 months | Secondary schools (n=2 in intervention arm)  Students aged 14-15 years (n=50 in intervention arm)  Teachers (n=not reported) | No theory reported | Dose | Attendance at programme sessions | Not reported | Student | Students attended 7 of 10 school sport sessions, 6 of 8 lunch-time sessions, 4 of 6 PA leadership sessions and 29 of the 50 students submitted their PA and nutrition handbooks | Not reported |
|  |  |  |  | Responsiveness | Programme satisfaction | Student questionnaire after study completion | Student | Overall, participants were satisfied with the programme | Not reported |
| Martens (2006) (44)  Krachtvoer  Netherlands | Programme consisting of classroom curriculum aimed to increase intake of fruit and fruit juice, decrease the consumption of high-fat snacks and increase breakfast frequency and quality  Duration 8 lessons within 3 months | Secondary school (n=11 in intervention arm)  Students aged 12-14 years (n=1021)  Teachers (n=15) | No theory reported | Adherence | Fidelity of implementation | Classroom observations at least one lesson | Teacher | 7 of the lessons were executed according to the curriculum | Not reported |
|  |  |  |  | Dose | Completeness of implementation | Structured monitoring reports for teachers after each lesson | Teacher | 8 teachers had between 4-8 lessons lasting 100 min each, 6 teachers had between 7-11 lessons lasting 50 min each and 1 teacher had 7 lessons lasting 60 min each | Not reported |
|  |  |  |  | Responsiveness | Students’ reaction to the programme | Student questionnaire after programme completion | Student | Students were moderately positive about the programme; mean score 7.2 | Not reported |
|  |  |  |  |  | Teachers opinion about the programme | Interview with teachers after programme completion | Teacher | Teachers had favourable opinions about the programme | Not reported |
| Muckelbauer (2009) (43)  School based programme for overweight prevention    Germany | Programme consisting of an education and environmental component aimed to promote drinking of water  Duration 1 school year | Primary schools (n=17)  Students aged 7-9 years (n=1744)  Teachers (n=85) | No theory reported | Dose | Not reported | Teacher questionnaire and structured interview 8 months after the start of the programme | Teacher | Teachers implemented on average 2.4 out of the four lessons | Not reported |
| Nanney (2011) (42)  School Breakfast Programme (SBP)  United States | Programme consisting of offering a healthy grab-n-go menu, halfway delivery service and eating in the classroom aimed to improve school breakfast participation  Duration 6 weeks | Primary Schools (n=1)  Students aged 10-11 years (n=239)  Teachers (n=10) | No theory reported | Responsiveness | Satisfaction with classroom eating | Student questionnaire after programme completion | Student | Majority of the Students were very satisfied with eating in the classrooms (64%) and with the taste of the healthy breakfast menu items | Not reported |
| Naylor (2006) & McKay (2014) (40, 94)  Action Schools! BC  Canada | Programme consisting of a ‘whole-school model’ focussing on school environment, scheduled PE, classroom action, family and community, extra-curricular and school spirit aimed to enhance PA  Duration 11 months | Primary schools (n=7 in intervention arm)  Students aged 9-12 years (n=515)  Teachers (n=42 in phase I and n=49 in phase II) | No theory reported | Dose | Fidelity | Teachers activity logs, weekly | Teacher | Teachers implementation was moderate (75%) | Beneficial effects on bone health were larger in schools where the compliance with the intervention was higher |
|  |  |  |  | Responsiveness | Satisfaction | Feedback surveys at the end of the study | Teacher | Teachers (91%) were highly satisfied with the components of AS! BC | Not reported |
| Naylor (2010) (41)  Action Schools! BC  Canada | Programme consisting of a whole-school model focussing on school environment, scheduled PE, classroom action, family and community, extra-curricular and school spirit aimed to enhance PA and HE  Duration 11 months | Primary schools (n=3)  Students aged 9-12 years (n=not reported)  Teachers (n=19) | No theory reported | Dose | Fidelity | Teachers activity logs, weekly | Teacher | Schools delivered an average of 140.1 min of PA opportunities per week. Scheduled PE was delivered on average 1.75 times per week, with a mean of 76.4 min. The average number of nutrition activities delivered per week was 2.27 with a mean of 15.6 min | Not reported |
| Perry (1997) & Heath (2002) (55, 93)  Coordinated Approach to Child Health (CATCH)  United States | Programme consisting of classroom curriculum, food services, targeting PE and families aimed to promote DB  Duration 3 years | Primary schools (n=50)  Students aged 8-11 years (n= not reported)  Teachers (n= not reported) | No theory reported | Dose | Implementation | Teacher questionnaire on one occasion | Teacher | There was a large range in use of CATCH activities by PE teachers, from 10% to 100% of classes taught with CATCH materials (average of 50% of classes taught having CATCH activities) | Not reported |
| Prins (2012) (39)  YouRAction  Netherlands | Programme consisting of computer-tailored intervention aimed to promote PA  Duration one year (3 lessons) | Secondary schools (n=31 classes in intervention arm)  Students aged 12-13 years (n=682 in intervention arm)  Teachers (n=not reported) | No theory reported | Dose | Exposure/use | Web server logs (log in frequency and the number of times an adolescent attempted to log-in and accessed the first page of each lesson) one month after programme completion | Student | Number of school lessons ranged from 2.46-2.63 and log in frequency ranged from 2.49-2.68 and 20-21% made one homework assignment | Not reported |
|  |  |  |  | Responsiveness | Appreciation | Student questionnaires one month after programme completion | Student | On a Likert scale from 1-5, scores ranged from 2.52-2.57 | Not reported |
| Reinaerts (2007) (38)  FV distribution programme versus a multicomponent programme  Netherlands | Programme consisting of free FV distribution, classroom curriculum, parental involvement and environmental component aimed to increase daily FV consumption  Duration 8 months | Primary schools (n=6 schools)  Students aged 4-12 years (n=not reported)  Teachers (n=60) | No theory reported | Adherence | Implementation: to what extent the 2 FV programmes were implemented by teachers | Teacher monitoring report during whole programme period | Teacher | Teachers had followed instruction manual largely or completely, ranging from 73% to 100% | Not reported |
|  |  |  |  | Dose | Implementation: to what extent the 2 FV programmes were implemented by teachers | Teacher monitoring report during the whole programme period | Teacher | Teachers had implemented most of the lessons but more in the first period than in the third period | Not reported |
|  |  |  |  | Responsiveness | Appreciation | Teacher questionnaire after programme completion | Teacher | Appreciation ranged from 7.1-8.7 | Not reported |
| Reynolds (2000) (90)  High 5 intervention  United States | Programme consisting of a classroom, family and cafeteria component aimed to increase FV intake  Duration during 2 school years | Primary schools (n=14 in intervention arm)  Students on average 8.7 years (n=1,698 in full trial)  Curriculum coordinators (n=9) | No theory reported | Adherence | Implementation: use of the materials | Coordinators' classroom checklist after each session, Coordinators' rating of the classroom curriculum after completion of the curriculum and classroom observations, timing not reported | Teacher | More than 90% of activities were implemented in each lesson | Not reported |
|  |  |  |  | Dose | Student attendance | Coordinators' attendance records, timing not reported | Student | Student attendance ranged from 93-96% | Not reported |
| Robbins (2014) (36)  Guys Only Active for Life (G.O.A.L.)  United States | Programme consisting of 15-min individual level motivational interviewing session during the school day by a registered (school) nurse and PA club aimed to encourage boys to attain adequate PA  Duration 7 weeks | Primary schools (n=1 in intervention arm)  Students aged 11-12 years (n=31 in intervention arm)  Nurses (n=1 and n=4 PA instructors) | Key process evaluation components defined by Steckler and Linnan | Adherence | Fidelity: the extent to which the intervention was implemented in the manner and spirit it was intended | Audio-taping motivational interviews, timing not reported | Nurse | The score indicated high fidelity related to session delivery and adherence to the underlying theories | Not reported |
|  |  |  |  | Dose | Dose delivered: what is actually delivered to participants and reflects the efforts or behaviours of the interventionists to provide the opportunity of the planned amount of intervention | Sessions times recorded on attendance sheets and data from 9 audio taped sessions at the beginning of the programme | Nurse | Every boy attended as scheduled. Duration of motivational interviewing sessions ranged from 6 min, 4s to 20 min, 39s and the average length was 13 min, 29s. Four sessions lasted fewer than 10 min | Not reported |
|  |  |  |  | Quality of delivery | Fidelity: the quality of intervention delivery | Audio-taping motivational interviews, timing not reported | Nurse | 8 of the 12 global dimensions ratings were above 4.0, indicating competency in motivational interviewing delivery | Not reported |
| Robbins (2012) (37)  Girls on the Move  United States | Programme consisting of face to face counselling sessions  aimed to elicit positive behaviour change in PA  Duration 6 months | Primary and secondary schools (n=1)  Students aged 11-13 years (n=37)  Nurses (n=1) | No theory reported | Adherence | Ensure that interventions are congruent with the underlying theory of behaviour change | Audio-taping motivational interviews, timing not reported | Nurse | Motivational interviewing global dimension element mean scores for the counselling sessions delivered by the school nurse at each of the 3 time points were all at the competency level of 4.00 or above | Not reported |
| Salmon (2005) (35)  ‘Switch-Play’ intervention  Australia | Programme consisting of classroom sessions  aimed to reduce the time spent in SB and increasing skills in, enjoyment of and participation in PA  Duration 19 lessons | Primary schools (n=8 in intervention arm)  Students aged 10 years (n=232 in 3 intervention arms)  Teachers (n=33) | No theory reported | Dose | Intervention quantity: how many children participated in the various lessons | Self-report of teachers each lesson | Teacher | Children completed 57 to 62% of the sheets | Not reported |
|  |  |  |  | Adherence | Intervention quality: programme delivered as intended | Self-report of teachers each lesson | Teacher | 92% of tasks undertaken in class were completed | Not reported |
|  |  |  |  | Responsiveness | Subjective evaluations of the interventions components | Self-report of teacher each lesson and student questionnaire after programme completion | Student | The children's enjoyment of the fundamental motor skills lessons was rated 4.5 and the behaviour modification lessons 4.4. A high proportion of children enjoyed the programme | Not reported |
| Salmon (2010) (34)  Switch-2-Activity  Australia | Programme consisting of classroom curriculum aimed to reduce SB and improve PA  Duration 6 lessons | Primary schools (n=8 in intervention arm)  Students aged 9-12 years (n=467 in intervention arm)  Teachers (n=33) | No theory reported | Dose | Exploration whether teachers delivered all aspects | One-in-one interviews after programme completion | Teacher | 71% of teachers delivered at least 4 of the 6 lessons | Not reported |
|  |  |  |  | Responsiveness | Enjoyment of the programme | Student questionnaires after programme completion | Student | Approximately 80% of children enjoyed the programme | Not reported |
| Saunders (2006) & Ward (2006) (26, 33)  Lifestyle Education for Activity Programme (LEAP)  United States | Programme consisting of changes in instructional practices and the school environment aimed to promote PA  Duration 2 years | Secondary schools (n=12)  Students aged 14-15 years (n=863 in intervention arm)  Teachers (n=23) | Key process evaluation components defined by Steckler and Linnan, Conceptual framework of process evaluations by Mcgraw and Logic model by Scheirer | Dose | Fidelity and completeness | Leap records consisting of record review, observation and a scoring list at two PA lessons per semester | Teacher | Workshops were given in 86.7% of the schools in year 1 and 70.8% of the schools in year 2. Overall school participation in service training and demonstration was 37.5%. 75% of the instructional elements were implemented across the 12 schools | Dose effect in proportions of participation in VPA from control, low implementers to high implementers |
| Shah (2011) (32)  SALSA programme  United States | Programme consisting of a video, games and empower activities  aimed to increase PA, consumption of FV and awareness about local healthcare services  Duration three separate school periods | Secondary Schools (n=1)  Students aged 13-14 years (n=not reported)  Peer leaders (n=16) | No theory reported | Adherence | Fidelity of the implementation strategies; quality assurance | Peer leader questionnaire  each lesson | Peer leader | Lessons were implemented as intended. However, there were some problems with maintaining class control and completing all activities with the given time limit | Not reported |
|  |  |  |  | Quality of delivery | Definition not described | Observations by teachers, timing not reported | Teacher | Peer leaders were prepared for their lessons and communicated the information well | Not reported |
|  |  |  |  | Responsiveness | Definition not described | Student questionnaire each lesson and observations, timing not reported | Student | The majority of the students had a genuine interest in the programme activities. Teachers indicated that students were interested in the topics and participated fully in all activities | Not reported |
| Sharma (2015) (31)  Brighter Foods  United States | Programme consisting of distribution of fresh FV, parent handouts, recipe demonstration and implementing a coordinated school health programme  aimed to provide access to fresh FV and nutrition education  Duration 16 weeks | Primary schools (n=1)  Students aged 9-10 years (n=57)  Teachers (n=3) | No theory reported | Dose | Dosage | Teacher questionnaire, timing not reported | Teacher | All (100 %) of the four 3rd grade teachers and one PE coach reported implementing CATCH classroom curriculum and PE components | Not reported |
| Singh (2009) (30)  Dutch obesity intervention in teenagers  Netherlands | Programme consisting of a classroom and environmental component aimed to improve EBRBs  Duration 8 months | Secondary schools (n=10 in intervention arm)  Students aged 12-13 years (n=632 in intervention arm)  Teachers (n=22) | RE-AIM framework by Glasgow | Adherence | Implementation | Teacher questionnaires after programme completion | Teacher | 50% of the teachers implemented the programme as described in the manual | Not reported |
|  |  |  |  | Responsiveness | Judgement/Satisfaction | Student questionnaires after programme completion | Student | Students rated the programme with a 6.6 (10-point scale) and the layout of the programme materials with a 3.5 (5-point scale) | Not reported |
|  |  |  |  |  |  | Teacher questionnaires after programme completion | Teacher | The majority of the teachers rated the content of the programme materials of the classroom programme as very good or good | Not reported |
| Story (2000) (29)  5-a-Day Power Plus  United States | Programme consisting of behavioural curricula in classroom, parental involvement, school food service modifications and industry support aimed to increase FV consumption  Duration 8 weeks | Primary schools (n=10)  Students aged 9-11 years (n=5,262 in grade 4 and n=5,324 in grade 5)  Teachers (n=31 in grade 4 and n=30 in grade 5) | No theory reported | Adherence | The percentage of the curriculum guide followed most/all the time | Teacher self-report, weekly and classroom observations each teacher on one taste-session and one lesson | Teacher | 83-92% of the teachers reported following the curriculum guide most or all the time. Observations found that 90-91% of lessons were implemented as planned | There were no significant differences in student FV intakes between low and high schools for process measures |
|  |  |  |  | Dose | The percentage of the entire lesson completed | Teacher self-reports, weekly | Teacher | Corresponding drop in adherence to following the curriculum guide was 94% to 85% in grade 4 and 93 to 78% in grade 5 |  |
| Van Nassau (2016) (28)  Dutch Obesity Intervention in Teenagers (DOiT)  Netherlands | Programme consisting of classroom, environment and parental component  aimed to increase awareness of EBRBs and increasing awareness and coping mechanisms for the influence of the (obesogenic) environment  Duration 2 school years | Secondary schools (n=20)  Students aged 12-14 years (n=1459)  Teachers (n=110) | Theory of diffusion of innovations by Rogers, Key process evaluation components defined by Steckler and Linnan, How to guide for developing a process evaluation by Saunders and RE-AIM framework by Glasgow | Adherence | Fidelity: the extent to which the teachers have implemented the programme as intended by the developers; compliance to the teacher manual | Teacher questionnaire after 8 and 20 months | Teacher | Fidelity to the teacher manual ranged from 85 to 26%. On average 56% of the lessons were delivered according the teacher manual | Implementation index score was a combination of the process measures fidelity, dosage and quality of delivery. Some evidence was found for a trend between a higher implementation index score and programme effectiveness |
|  |  |  |  | Dose | Dosage; the proportion of the lessons that were actually delivered or performed by the teachers and received by the adolescents | Teacher questionnaire after 8 and 20 months | Teacher | On average teachers delivered 3.5 (year1) and 2.2 (year2) of the 6 lessons per year. 22% distribute the parent information, 27% had given homework and 17% send parent newsletter |  |
|  |  |  |  | Quality of delivery | Quality of delivery | Teacher questionnaires after 8 and 20 months | Teacher | No results |  |
|  |  |  |  | Responsiveness | Satisfaction with the programme and materials | Teacher questionnaire after 8 eight and 20 months | Teacher | Overall teachers were satisfied with the programme and materials | Not reported |
|  |  |  |  |  |  | Student questionnaire after 20 months | Student | Students were moderately satisfied with the materials | Not reported |
| Verloigne (2015) (91)  IDEFICS  Belgium | Programme consisting of 10 modules at community and school level aimed to increase PA  Duration 2 years | Preschool and primary schools (n=34)  Students aged 2-9.9 years (n=976)  Teachers and school working groups (n=not reported) | Key process evaluation components defined by Steckler and Linnan and  How to guide for developing a process evaluation by Saunders | Dose | Process evaluation score | Teacher questionnaire, weekly | Teacher | The mean intervention process score was 20.9 (max=44) | The decrease in PA and increase in sedentary time was higher in low implementing schools, compared to medium and high implementing schools |
| Wang (2010) (27)  Comprehensive school intervention  United States | Programme consisting of integration of nutrition and food systems concepts into academic curriculum and cooking and gardening programmes  aimed to transform school foodservices and dining experiences  Duration 3 years | Primary Schools (n=4)  Students aged 9-11 years (n=327)  Teachers (n=18) | No theory reported | Dose | Student exposure to the intervention | Student questionnaire after first 2 years of implementation | Student | More than one-third of the students not ever had a cooking class, and only 15% of the students not ever had a gardening class. About 18% and 28% of the students attended a cooking class and a gardening class for 5 years or more | Students most exposed to the programme increased their consumption of FV by 0.5 cups (one standard serving) while students least exposed decreased their consumption by 0.3 cups. Students most exposed to the programme also showed a significantly greater increase in preference for fruit and green leafy vegetables, compared to students least exposed to the programme |
| Wind (2007) (25)  Pro Children  Norway, Spain and the Netherlands | Programme consisting of a multicomponent intervention aimed to promote the consumption of FV  Duration 7 months (16 lessons) | Primary and Secondary schools (n=62)  Students aged 10-13 years (n=868)  Teachers (n=818) | No theory reported | Adherence | Quality (fidelity) | Teacher questionnaire after programme completion | Teacher | Total curriculum implementation score was 9.1 (combined score of quality and quantity) | Implementation score was significantly associated with FV intake |
|  |  |  |  | Dose | Quantity (dose delivered) |  |  |  |  |
|  |  |  |  | Responsiveness | Appreciation | Student questionnaire after programme completion | Student | Mean score on appreciation was 2.3 | Appreciation of the project was significantly associated with FV intake |

Abbreviations: PA = physical activity; FV = fruit and vegetables; PE = physical education; HE = healthy eating; SB = sedentary behaviour; EBRBs= energy balance-related behaviours; DB = dietary behaviour; min = minutes
