# Supplementary material for: Measuring implementation fidelity of school-based obesity prevention programmes: a systematic review
Source: Int J Behav Nutr Phys Act. 2018 Aug 13;15:75. doi: 10.1186/s12966-018-0709-x (PMC6088402; doi:10.1186/s12966-018-0709-x)
Supplement: Supplementary file 4 — Quality assessment. Full overview of the quality assessment of the fidelity components. (DOCX 48 kb) [file 12966_2018_709_MOESM4_ESM.docx]

**Table 4. Quality assessment**

|  | **Criterion** | **1** | **2** | **3** | **4** | **5** | **6** | **7** | **Validity score** |
| --- | --- | --- | --- | --- | --- | --- | --- | --- | --- |
| Alaimo (2015) (78) | dose | - | - | + | - | - | + | - | 29% |
| Almas (2013) (76) | dose | - | - | + | - | - | - | - | 14% |
|  | responsiveness | - | - | - | - | + | - | - | 14% |
| Aarenstrup (2015) & Jørgensen (2015, 2017) (51, 77, 87) | adherence | + | NA | + | - | + | + | - | 67% |
|  | dose | + | + | + | - | + | + | + | 86% |
|  | responsiveness | + | + | + | - | - | - | - | 43% |
| Barr-Anderson (2012) (75) | dose | - | - | + | - | + | + | - | 43% |
|  | quality of delivery | - | NA | + | - | + | + | - | 50% |
|  | responsiveness | - | + | + | + | + | + | - | 71% |
| Battjes-Fries (2016) (79) | dose | - | - | + | - | + | - | + | 43% |
|  | responsiveness | - | + | + | - | + | - | + | 57% |
| Bere (2005) (74) | dose | - | - | - | - | + | + | + | 43% |
|  | responsiveness | - | - | - | - | + | + | + | 43% |
| Bergh (2012) (73) | dose | - | - | + | - | + | + | + | 57% |
| Bessems (2011 & 2013) (71, 72) | adherence | - | NA | + | - | + | + | - | 50% |
|  | dose | - | - | + | - | + | + | + | 57% |
|  | responsiveness | - | + | - | - | + | - | - | 29% |
| Blaine (2017) (80) | dose | + | - | + | - | + | + | - | 57% |
|  | responsiveness | + | - | + | - | + | + | - | 57% |
| Blom-Hofman (2004) (69) | adherence | - | NA | + | - | - | + | - | 33% |
|  | responsiveness | - | + | - | - | + | - | - | 29% |
| Blom-Hoffman (2008) (70) | adherence | - | NA | - | + | - | + | - | 33% |
|  | dose | - | - | - | - | - | - | - | 0% |
|  | responsiveness | - | + | - | - | - | - | - | 14% |
| Burgermaster (2017) (81) | adherence | - | NA | - | - | + | + | + | 50% |
|  | quality of delivery | - | NA | + | - | + | + | + | 67% |
|  | responsiveness | - | - | - | - | + | - | + | 29% |
| Campbell (2015) (68) | adherence | - | NA | - | + | + | + | - | 50% |
|  | dose | - | - | + | - | + | + | - | 43% |
| Christian (2012) (67) | dose | - | - | - | - | - | + | + | 29% |
|  | responsiveness | - | + | - | - | - | + | + | 43% |
| Dalton (2014) (66) | dose | - | - | + | - | - | + | - | 29% |
|  | responsiveness | - | - | + | - | - | + | - | 29% |
| Davis (2000) (65) | dose | - | - | - | + | + | + | - | 43% |
| Davis (2003) & Steckler (2003) (21, 64)21) | dose | - | + | + | + | - | + | - | 57% |
| Day (2008) & Naylor (2016) (63, 92) | dose | - | - | - | - | + | + | - | 29% |
|  | quality of delivery | - | NA | - | - | + | - | - | 17% |
|  | responsiveness | + | - | - | - | + | - | - | 29% |
| De Meij (2013) (62) | dose | + | - | + | + | - | + | - | 57% |
| Dubuy (2014) (82) | dose | - | - | - | - | - | - | - | 0% |
|  | responsiveness | - | - | - | - | - | - | - | 0% |
| Dunton (2009) (61) | adherence | + | NA | + | - | - | + | - | 50% |
|  | dose | + | - | + | - | - | - | - | 29% |
| Dunton (2014) (60) | adherence | + | NA | + | + | + | + | - | 83% |
|  | dose | + | - | - | - | + | - | - | 29% |
| Eather (2016) (83) | dose | - | - | - | - | - | + | - | 14% |
|  | responsiveness | - | - | - | - | + | - | - | 14% |
| Elinder (2012) (59) | adherence | - | NA | + | - | - | - | - | 17% |
| Ezendam (2013) (58) | dose | + | - | + | + | + | + | + | 86% |
|  | responsiveness | + | - | - | - | + | - | + | 43% |
| Gibson (2008) (57) | dose | + | - | + | + | - | + | - | 57% |
|  | quality of delivery | + | NA | - | - | + | - | - | 33% |
|  | responsiveness | + | - | - | - | - | + | - | 29% |
| Griffin (2017) (86) | adherence | + | NA | - | + | - | + | - | 50% |
| Hankonen (2017) (84) | responsiveness | - | + | - | - | + | + | - | 43% |
| Harris (1998) (85) | adherence | - | NA | - | - | - | + | - | 17% |
| Hildebrand (2012) (54) | dose | - | - | - | - | - | - | - | 0% |
| Jan (2009) (53) | responsiveness | - | - | - | - | + | - | - | 14% |
| Janssen (2013) (52) | dose | + | - | + | + | + | + | - | 71% |
|  | responsiveness | + | - | + | - | + | + | - | 57% |
| Jurg (2006) (50) | dose | - | - | + | + | - | - | - | 29% |
| King (2014) and Lederer (2015) (47, 49) | adherence | - | NA | + | + | + | - | - | 50% |
| Lane (2017) (88) | responsiveness | - | + | - | - | - | + | - | 29% |
| Larsen (2015) (48) | adherence | + | NA | + | + | + | + | - | 83% |
|  | dose | + | - | + | + | + | + | - | 71% |
|  | responsiveness | + | - | + | - | + | - | - | 43% |
| Lee (2013) & Gray (2015) (46, 56) | adherence | + | NA | + | - | + | + | + | 83% |
|  | dose | + | - | + | - | + | + | + | 71% |
|  | responsiveness | + | - | + | + | + | + | + | 86% |
| Lehto (2014) (45) | dose | - | - | - | - | + | - | + | 29% |
| Levine (2002) (24) | adherence | - | NA | - | - | - | + | - | 17% |
|  | dose | - | - | - | - | - | + | - | 14% |
| Little (2015) (89) | dose | - | - | + | + | - | + | + | 57% |
|  | quality of delivery | - | NA | + | + | - | + | - | 50% |
|  | responsiveness | - | + | + | + | - | + | - | 57% |
| Lubans (2011) (23) | dose | - | - | + | - | - | + | - | 29% |
|  | responsiveness | - | - | - | - | + | - | - | 14% |
| Martens (2006) (44) | adherence | - | NA | - | - | - | + | - | 17% |
|  | dose | - | - | - | + | - | + | - | 29% |
|  | responsiveness | - | + | + | - | - | - | - | 29% |
| Muckelbauer (2009) (43) | dose | - | - | - | + | - | - | - | 14% |
| Nanney (2011) (42) | responsiveness | - | - | - | - | + | - | - | 14% |
| Naylor (2006) & McKay (2014) (40, 94) | dose | - | - | - | - | - | + | + | 29% |
|  | responsiveness | - | - | - | - | + | - | - | 14% |
| Naylor (2010) (41) | dose | - | - | - | - | - | + | - | 14% |
| Perry (1997) & Heath (2002) (55, 93) | dose | - | - | - | - | - | - | - | 0% |
| Prins (2012) (39) | dose | - | - | - | + | + | - | - | 29% |
|  | responsiveness | - | - | - | - | + | - | - | 14% |
| Reinaerts (2007) (38) | adherence | - | NA | + | - | + | + | - | 50% |
|  | dose | - | - | + | - | + | + | - | 43% |
|  | responsiveness | - | - | - | - | + | - | - | 14% |
| Reynolds (2000) (90) | adherence | - | NA | - | + | - | + | - | 33% |
|  | dose | - | - | - | - | - | + | - | 14% |
| Robbins (2014) (36) | adherence | + | NA | + | - | + | + | - | 67% |
|  | dose | + | - | + | - | + | + | - | 57% |
|  | quality of delivery | + | NA | + | - | + | + | - | 67% |
| Robbins (2012) (37) | adherence | - | NA | + | - | + | + | - | 50% |
| Salmon (2005) (35) | adherence | - | NA | + | - | - | + | - | 33% |
|  | dose | - | - | + | - | - | + | - | 29% |
|  | responsiveness | - | - | + | + | - | + | - | 43% |
| Salmon (2010) (34) | dose | - | - | + | - | - | - | - | 14% |
|  | responsiveness | - | - | + | - | - | - | - | 14% |
| Saunders (2006) & Ward (2006) (26, 33) | dose | + | - | - | + | + | + | + | 71% |
| Shah (2011) (32) | adherence | - | NA | - | - | - | + | - | 17% |
|  | quality of delivery | - | NA | - | - | - | + | - | 17% |
|  | responsiveness | - | + | - | + | - | + | - | 43% |
| Sharma (2015) (31) | dose | - | - | - | - | - | - | - | 0% |
| Singh (2009) (30) | adherence | + | NA | - | - | - | - | - | 17% |
|  | responsiveness | + | + | - | - | - | - | - | 29% |
| Story (2000) (29) | adherence | - | NA | + | + | - | + | + | 67% |
|  | dose | - | - | + | - | - | + | + | 43% |
| Van Nassau (2016) (28) | adherence | + | NA | + | - | + | + | + | 83% |
|  | dose | + | - | + | - | + | + | + | 71% |
|  | quality of delivery | + | NA | + | - | - | + | + | 67% |
|  | responsiveness | + | + | + | - | + | + | - | 71% |
| Verloigne (2015) (91) | dose | + | - | - | - | + | + | + | 57% |
| Wang (2010) (27) | dose | - | - | + | - | - | - | + | 29% |
| Wind (2007) (25) | adherence | - | NA | - | - | + | - | + | 33% |
|  | dose | - | - | - | - | + | - | + | 29% |
|  | responsiveness | - | - | - | - | + | - | + | 29% |

Rating of criteria: + = yes, - = no | NA = Not applicable | Criteria: 1.Model used for evaluation; positive if a theoretical framework or model for the evaluation was used and reported or referred to in the article, negative if no theoretical framework or model was used for the evaluation. 2.Level of evaluation; positive if the fidelity component was evaluated on two or more levels (i.e. school director, teacher, student), negative if the fidelity component was evaluated on only one level (i.e. school director, teacher, student) *. 3.Operationalisation of fidelity component; positive if the fidelity component was defined or operationalised, negative if only the name of the fidelity component was provided and not further defined or operationalised. 4.Data collection methods; positive if two or more techniques for data collection were used (triangulation), negative if only one technique for data collection was used. 5.Quantitative fidelity measures; positive if measurement of the fidelity component was performed with adequately described methods, negative if measurements of the fidelity component was not performed with adequately described methods **. 6.Frequency of data collection; positive if the fidelity component was measured on more than 1 occasion (e.g. pre, during after delivery), negative if the fidelity component was measured on only 1 occasion. 7. Relation fidelity component and programme outcome assessed; positive if tested whether the fidelity component was related to programme outcomes, negative if not tested whether the fidelity component was related to programme outcomes.| * only applied to dose, responsiveness and differentiation, as it is not possible to evaluate adherence and quality of delivery on two or more levels – i.e. only on teacher level. | ** adequate = sufficient information to be able to repeat the study.
